# High-resolution profiles of the *Streptococcus mitis* CSP signaling pathway reveal core and strain-specific regulated genes

**DOI:** 10.1186/s12864-018-4802-y

**Published:** 2018-06-13

**Authors:** G. Salvadori, R. Junges, H. A. Åmdal, T. Chen, D. A. Morrison, F. C. Petersen

**Affiliations:** 1 Institute of Oral Biology, Faculty of Dentistry, University of Oslo, Postboks 1052, Blindern, 0316 Oslo, Norway; 2 Department of Microbiology, The Forsyth Institute, Cambridge, MA USA; 3 Department of Biological Sciences, College of Liberal Arts and Sciences, University of Illinois at Chicago, Chicago, IL USA

**Keywords:** *Streptococcus mitis*, Natural transformation, CSP, Competence, Quorum sensing

## Abstract

**Background:**

In streptococci of the mitis group, competence for natural transformation is a transient physiological state triggered by competence stimulating peptides (CSPs). Although low transformation yields and the absence of a widespread functional competence system have been reported for *Streptococcus mitis*, recent studies revealed that, at least for some strains, high efficiencies can be achieved following optimization protocols. To gain a deeper insight into competence in this species, we used RNA-seq, to map the global CSP response of two *transformable strains*: the type strain NCTC12261^T^ and SK321.

**Results:**

All known genes induced by ComE in *Streptococcus pneumoniae*, including *sigX*, were upregulated in the two strains. Likewise, all sets of streptococcal SigX core genes involved in extracellular DNA uptake, recombination, and fratricide were upregulated. No significant differences in the set of induced genes were observed when the type strain was grown in rich or semi-defined media. Five upregulated operons unique to *S. mitis* with a SigX-box in the promoter region were identified, including two specific to SK321, and one specific to NCTC12261^T^. Two of the strain-specific operons coded for different bacteriocins. Deletion of the unique *S. mitis sigX* regulated genes had no effect on transformation.

**Conclusions:**

Overall, comparison of the global transcriptome in response to CSP shows the conservation of the ComE and SigX-core regulons in competent *S. mitis* isolates, as well as species and strain-specific genes. Although some *S. mitis* exhibit truncations in key competence genes, this study shows that in transformable strains, competence seems to depend on the same core genes previously identified in *S. pneumoniae*.

**Electronic supplementary material:**

The online version of this article (10.1186/s12864-018-4802-y) contains supplementary material, which is available to authorized users.

## Background

In several streptococci intercellular coordination of gene expression mediated by peptide pheromones is associated with development of competence for transformation [1]. The pheromones activate a signal transduction pathway that regulates natural transformation. This physiological feature provides a selective advantage by allowing competent cells to acquire new characteristics, such as antibiotic resistance, by incorporation of DNA from other cells. In *Streptococcus pneumoniae*, natural competence is a tightly controlled transient state: it spontaneously arises during the early exponential growth phase at a certain cell density and reaches its peak after approximately 20 min, before it quickly shuts down [1, 2]. The regulatory cascade is induced via activation of *comC*, encoding the competence-stimulating peptide (CSP) [1], which is further cleaved and exported by its secretion apparatus ComAB [3]. CSP binds and activates its cognate receptor ComD, which, after phosphorylation, activates the response regulator ComE [4]. Phosphorylated ComE then specifically binds to a target conserved sequence, referred to as the ComE-box, in the promoter region of *sigX,* a global transcriptional modulator, in addition to *comCDE* itself and *comAB,* creating a positive feedback loop that coordinates an explosive spread of competence among nearby cells*.* These genes form the core of the quorum sensing apparatus for the induction of competence [5, 6] and are known as ‘early’ genes. SigX then initiates the transcription of a number of late genes involved in DNA uptake, recombination and fratricide by recognizing a SigX-box consensus sequence (TACGAATA) in their promoter regions [7, 8].

Many of the SigX-regulated genes contribute to various aspects of transformation. A dozen genes in four operons are required for assembly of the DNA transport machinery, while five genes in five operons are considered essential for efficient recombination of donor DNA strands [9]. Competent pneumococcal cells also upregulate at least six genes involved in the production of killing factors and their respective immunity proteins, including the early gene *comM*, and late genes *cibABC*, *cbpD* and *lytA* [10, 11]*.* CbpD, more specifically, is a murein hydrolase that plays a key role in the fratricide phenomenon, in which competent cells are able to kill and lyse non-competent sibling cells in the neighboring milieu, in a predatory mechanism most likely wired to acquire their DNA [11]. Interestingly, fratricide is not unique to pneumococci, as it has also been demonstrated in closely related species such as *Streptococcus mitis* and *Streptococcus oralis* [10].

*S. mitis* is a pioneer colonizer of the oral cavity, residing on the teeth, tongue and mucous membranes, as well as tonsils and nasopharynx, where it may exist side-by-side in biofilms with *S. pneumoniae* [12–14]. Both *S. mitis* and *S. pneumoniae* are naturally competent for natural transformation, and the exchange of genetic material by homologous recombination between them is recognized as part of their parallel evolution [15]. In addition, transfer of both antimicrobial resistance and virulence genes has been described between the species, evidenced by the presence of mosaic structures in gene sequences [16–18]. Interestingly, it has been suggested that the acquistion of *S. mitis* genes by *S. pneumoniae* has contributed to its evolution, but the opposite has not been proven [15]. Both species share a common ancestor, and it has been suggested that *S. mitis* evolved from a *S. pneumoniae* -like precursor by genome reduction, losing virulence genes and developing mechanisms of adaptation to the human host [15]. *S. mitis* strains represent a wide range of very distinct lineages under the same name, and previous reports have demonstrated that the genetic variability among *S. mitis* strains can be greater than between *S. mitis*, *S. pneumoniae* and *Streptococcus pseudopneumoniae* [19, 20]. Due to its ability to induce oral mucosal antibodies, *S. mitis* has been investigated for its potential as a vaccine vector [21]. Among 18 *S. mitis* strains that have been partially or completely sequenced, the majority of competence genes are widely conserved, with the exception of *sigX*, which appears in truncated forms in 44% of the strains analyzed [15, 22]. However, the activity, regulation, and possible role of *S. mitis* competence in the flow of genetic information between commensal and pathogenic streptococci remains unknown [22, 23]. Although low transformation yields and the absence of a widespread functional competence system have been reported for this species, recent studies reveal that, at least for some strains, high efficiencies can be achieved following optimization steps in current protocols [22]. Here, we applied such optimal conditions to gain the first detailed insight into the regulatory pathways of competence development and investigate the strain specificity of the CSP signaling response in two *S. mitis* isolates.

## Results

### *S. mitis* transformation efficiency in response to CSP

**Fig. 1:**
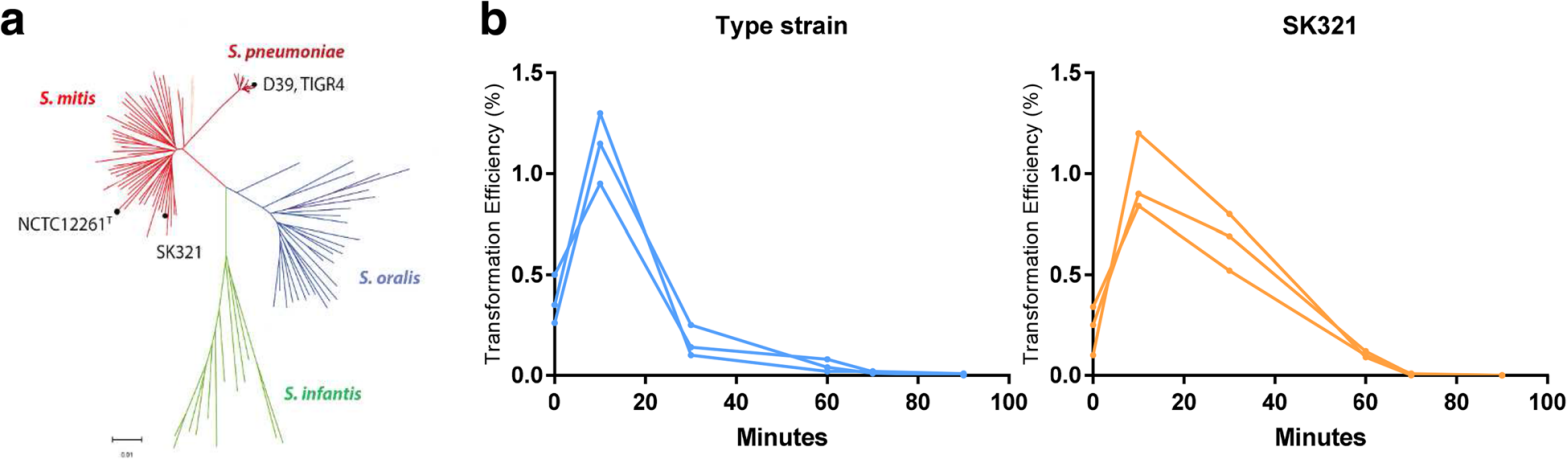
**a** Phylogenetic tree illustrating the separation of *S. mitis* NCTC12261^T^ and SK321 in different branches (adapted from [20]). **b** Kinetics of transformation in the *S. mitis* type strain and strain SK321. Pre-cultures at OD600 0.5 were diluted 1:100 in C + Y_YB_ medium and grown until OD600 0.04 at 37 °C in 5% CO_2_. Cultures were treated with 300 nM cognate CSP and distributed into 200 μL aliquots that were further exposed to 1 μg ml^− 1^ recombinant plasmid pVA838 at indicated times. 20 U ml^− 1^ DNase I were added after 30 min of exposure to DNA and the culture was incubated in air at 37 °C for additional 30 min. Transformants were recovered in blood agar plates supplemented with Erythromycin. Each line represents results of a single experiment

In contrast to *S. pneumoniae*, most *S. mitis* strains produce different strain-specific CSPs but seem to transform with low efficiency under laboratory conditions. Besides responding to different strain-specific stimulating peptides [22], the *S. mitis* type strain and strain SK321 are located phylogenetically apart (Fig. 1a) [20]. Pairwise comparison of the two strains performed by orthologous clustering using OrthoVenn revealed that the type strain has 1636 annotated proteins, whereas SK321 has 1757. One thousand, three hundred and seventy-nine orthologous clusters are shared, while 203 predicted proteins in the type strain and 352 in SK321 are strain-specific. Altogether, these features make the two *S. mitis* strains an interesting sample of *S. mitis* diversity for initial study of competence gene regulation in this species.

Competence for genetic transformation in pneumococcus occurs during a brief period of highly specialized protein synthesis (10–20 min), coordinated among many or all cells of an actively growing culture. Early genes present strongly increased expression during the period between 5 and 10 min after CSP induction, decreasing nearly to original values by 20 min after initiation of exposure to CSP. “Late” genes display a similar expression pattern, but with a delay of approximately 5 min [9]. Consistent with these timings, transformation kinetics for DNA uptake and recombination in the pneumococcus peaks between 10 to 15 min after CSP induction [24–26]. Evaluation of *S. mitis* type strain and SK321 temporal transformation patterns showed a remarkably similar pattern, with maximal transformation yields after 15 min of CSP induction in both strains (Fig. 1b). Interestingly, while transformation declined substantially after 30 min for the type strain, it declined more slowly in SK321. Based on these data, we chose 15 min after CSP induction as a suitable sampling time for evaluating competence-related gene expression.

### Responses to CSP by the *S. mitis* type strain and strain SK321

The competence response in *S. pneumoniae*, which has been described in detail in the literature, comprises three phases, early, late and delayed, which vary in magnitude depending on environmental conditions such as pH, temperature, and presence of albumin. While the early and late responses depend on well-defined regulons, the delayed response is less well understood, with no specific regulatory mechanisms yet identified. Thus, to better characterize the competence response in *S. mitis*, we investigated the transcriptome profile of the type strain in response to CSP during growth in two contrasting media, the semi-defined medium C + Y_YB,_ and the rich medium TSB. The medium C + Y_YB_ is an optimal medium for competence development for *S. mitis* type strain, supporting higher levels of *sigX* expression when compared to rich medium (TSB) or semi-defined C + Y [22].

**Fig. 2:**
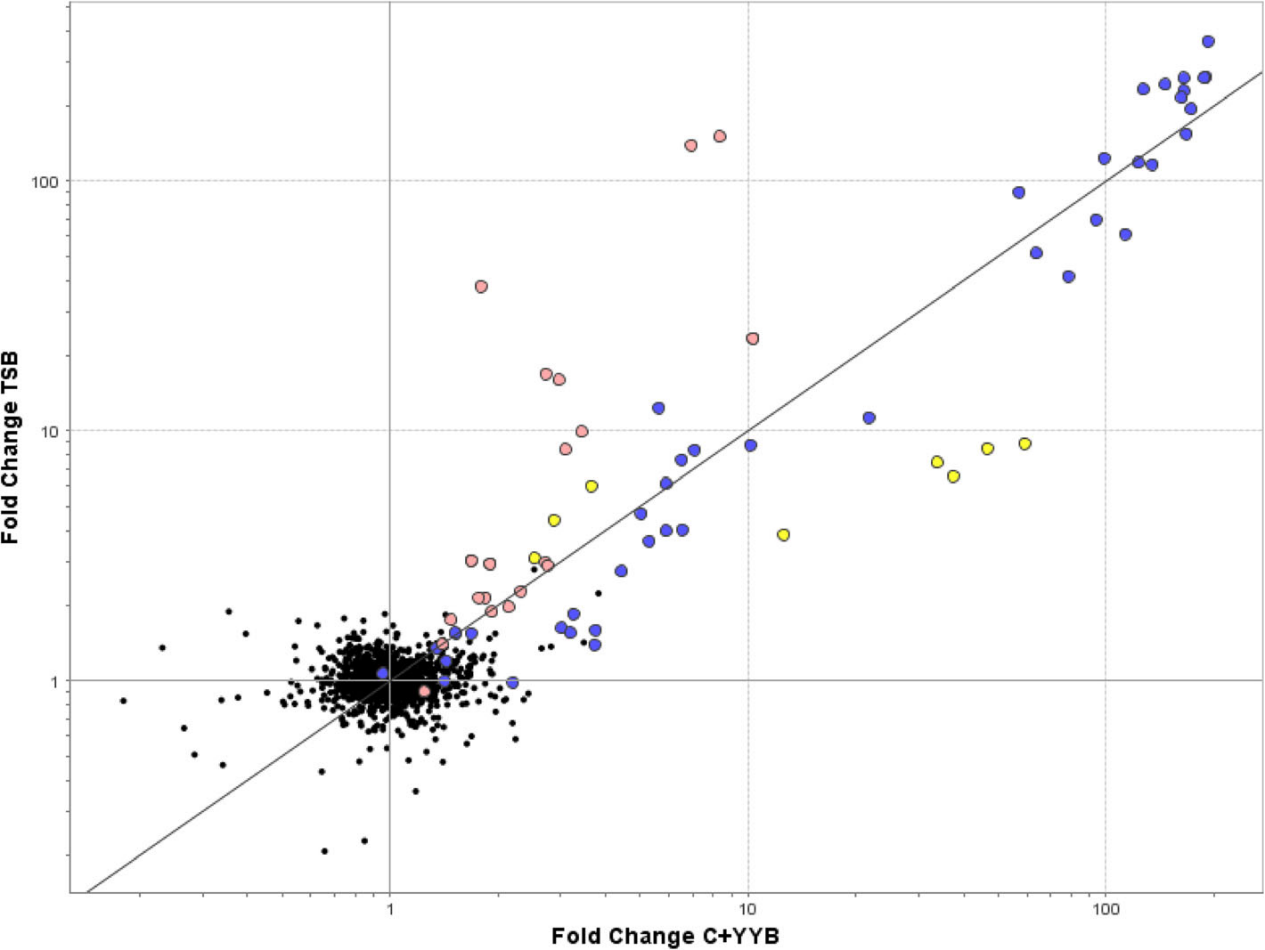
Correlation between gene expression changes induced by different growth media (TSB and C + Y_YB_) in *S. mitis* type strain. Fold changes values are for all significantly induced ORF sequences of *S. mitis* type strain genome and represent mean values for comparisons of CSP-treated and untreated samples from two independent biological experiments. Circles corresponding to genes under ComE regulation are represented in light pink, whereas genes under SigX regulation are represented in purple. Yellow circles correspond to significantly upregulated transcripts that do not present either ComE or SigX regulatory sites

In TSB, exposure to CSP in the type strain resulted in the upregulation of 68 genes by > 2-fold, while downregulating 21 genes (Additional file 1: Table S1). When C + Y_YB_ medium was used to examine the CSP response of *S. mitis* type strain, 79 genes were upregulated > 2-fold, whereas 19 were downregulated (Additional file 2: Table S2). There was a strong similarity in the response to CSP in different media with regards to the number of transcripts upregulated, with an overlap of 53 genes. Interestingly, among the downregulated genes, only two hypothetical proteins coincided between TSB and C + Y_YB_ media. With the exception of two gene clusters corresponding to the yellow circles in Fig. 2, the CSP response profile of *S. mitis* was only slightly affected by the choice of growth medium (Fig. 2). Three genes in one cluster are orthologues of delayed genes in *S. pneumoniae* (SP0785, SP0786 and SP0787) [9], and the other 5-gene cluster highly upregulated in C + Y_YB_ corresponds to a region that is not involved in competence in *S. pneumoniae*. The ComE regulon presented a modestly higher upregulation when exposed to TSB, whereas the SigX regulon seemed to respond similarly in both media. This difference indicates that environmental factor may be important for the regulatory processes in *S. mitis* competence.

In SK321, a strain grouped in a different cluster than the type strain, evaluation of the CSP-induced response was performed using the same cut-off values (> 2-fold). One hundred and sixty-seven genes positively responded to CSP in the strain SK321 when cultured in C + Y_YB_, while 69 showed significant downregulation (Additional file 3: Table S3). Overall, this isolate presented significantly higher expression levels than the type strain. This was not an isolated observation, since a similarly exacerbated response has been reported, as determined by means of luciferase reporter assays for the *sigX* gene [22]. For comparison of the two *S. mitis* isolates’ expression patterns, we will refer below only to data collected in C + Y_YB_ for both strains.

Since samples for RNA-seq were collected at a single time point (15 min post CSP treatment), it was not possible to classify gene expression by temporal response. However, given the genetic proximity of *S. mitis* to *S. pneumoniae* and similarity in temporal patterns of transformation, we searched for orthologues of differentially expressed genes in a competent *S. pneumoniae* strain derivative of R6 [27] and analyzed their promoter regions for conserved regulatory sequences. By doing so, it was possible to identify orthologous genes, their functions and the expression pattern obtained in this transcriptome analysis, and compare with the DNA microarray data already known for *S. pneumoniae*.

### Early response: Conservation of the ComE regulon

Genes upregulated by CSP in the *S. mitis* type strain and SK321 are listed with their orthologous pneumococcal early genes in Table 1. Orthologues of all *S. pneumoniae* ComE-responsive genes acting in regulation of the competence cascade and fratricide were identified as upregulated in both strains. These included 9 transcriptionally activated regions (TARs) accounting for a total of 21 genes responsible for CSP processing and export (*comAB*), competence regulators (*comCDE*) and *sigX* (duplicate copy), and bacteriocin related genes. An early gene encoding an immunity protein involved in competence-regulated self-protection (*comM*) was also upregulated, as well as several other orthologues of *S. pneumoniae* early genes, such as *comW, purA*, and *lytR* [9]. The only observable difference in the early response between the two *S. mitis* strains was the upregulation of the orthologue of SP2156 in SK321 (SMSK321_1581), a gene encoding a membrane bound protein of unknown function.

**Table 1:** *S. mitis* type strain and SK321 upregulated orthologues of *S. pneumoniae* early genes in response to CSP in C + Y_YB_

Gene ID			Mean fold-change^c^			
TIGR4^b^	NCTC12261^a^	SK321^a^	NCTC12261	SK321	R6^d^	Description
Orthologues of early genes						
0014	0014	1251	3.4	71.2	↑	Competence-specific global transcription modulator; *sigX1*
0018	0016	0337	1.8	10.8	↑	Conserved hypothetical protein; *comW*
0019	0017	0338	1.8	6.5	↑	Adenylosuccinate synthetase; *purA*
0042	0048	1310	8.3	41.6	↑	ABC transporter CbaT; *comA*
0043	0049	1311	6.9	48.3	↑	Transport protein ComB; *comB*
2006	0613	1651	3.1	36.3	↑	Competence-specific global transcription modulator; *sigX2*
2237	0788	1635	1.7	3.58	↑	Hypothetical protein; ComC
2236	0787	1634	1.8	18.9	↑	ComD
2235	01116^e^	00428^e^	1.7	19.3	↑	ComE
1110	0833	0680	1.9	10.9	↑	Riboflavin biosynthesis protein RibF; *ribF*
1945	0911	1653	10.3	75.4	↑	Lipoprotein. putative; *comM*
1944	0912	1654	2.3	10.9	↑	Conserved hypothetical protein
1943	0913	1655	2.1	8.0	↑	Acetyltransferase, GNAT family
1942	0914	1656	1.9	6.9	↑	Membrane-bound protein LytR
1717	0940	1696	2.7	12.6	↑	ABC transporter, ATP-binding protein
1716	0941	1697	3.0	12.6	↑	ABC transporter
1549	1162	0137	2.7	17.1	↑	Peptide deformylase
1548	1163	0138	2.8	16.8	↑	Conserved hypothetical protein
–	1164	0139	1.5	22.0	–	Conserved hypothetical protein
1547	1165	0140	1.4	4.6	–	Conserved hypothetical protein
2156	0729	1581	1	2	↑	SPFH domain-containing protein

^a^Gene number and product from HOMD

^b^Gene number and product from [51]

^c^Mean fold-change induction of CSP in *S. mitis* type strain and SK321 obtained by transcriptome analysis

^d^Expression pattern during response the of *S. pneumoniae* to CSP pheromone [9]

^e^Gene number according to PROKKA annotation [52]

In *S. pneumoniae*, early genes are preceded by a conserved regulatory sequence consisting of two 9 bp imperfect direct repeats (DRs) separated by 12 nucleotides (aCAnTTcaG-12-aCAgTTgaG), which is recognized as the phosphorylated ComE-binding site or ComE-box [8]. Alignment of the regions immediately upstream of the orthologues of early genes in the *S. mitis* type strain and SK321 revealed the presence of consensus motifs in all cases, except for the orthologue of SP2156 in the type strain (SM12261_0729), which was not upregulated by CSP (Table 2). During the search for DRs among the upregulated sequences, we noticed a ComE-box upstream of SMSK321_0027, which encodes an uncharacterized Gly-Gly peptide. This gene is orthologous to SP0429, which has never been associated to competence in *S. pneumoniae*,  most likely due to a defective regulatory sequence without the first 9 bp DR (Table 2). However, in our observation of transcriptome analysis of *S. pneumoniae* D39 (data not shown) endogenously competent cells showed upregulation of SPD_0391, orthologue of SMSK321_0027. We also detected a DR upstream of SPD_0391; interestingly, this gene has not been related to competence in D39 in a previous report [28]. Although carrying sequences slightly divergent from the consensus for DRs (Table 2), the gene encoding *comW* presented upregulation in both strains independent of medium composition. This suggests that this regulatory site is still responsive despite the presence of a less conserved DR.

**Table 2:** Alignment of putative ComE box sequences upstream of clusters of *S. mitis* type strain and SK321 orthologues of early genes

Function in competence	Annotation	Gene locus^a^	Consensus^b^				
			aCAnTTcaG	12	aCAgTTgaG		−10 site
Regulation	*comA*	SP0042	G**CA**G**TT**GG**G**	12	T**CA**T**TT**GG**G**	32	TAAGAT
		SM12261_0048	G**CA**T**TT**GG**G**	12	G**CA**T**TT**GG**G**	32	TAAGAT
		SMSK321_1310	G**CA**G**TT**GG**G**	12	G**CA**T**TT**GG**G**	32	TAAGAT
	*comC*	SP2237	A**CA**C**TT**TG**G**	12	A**CA**G**TT**GA**G**	31	TATAAT
		SM12261_0788	A**CA**C**TT**GG**G**	12	A**CA**G**TT**GA**G**	31	TATAAT
		SMSK321_5’1634	A**CA**C**TT**GG**G**	12	A**CA**G**TT**GA**G**	31	TATAAT
	*comW*	SP0018	C**CA**T**TT**TT**G**	10	G**CA**C**TT**AAa	38	TATACT
		SM12261_0016	Cttt**TT**GA**G**	12	A**CA**A**TT**CA**G**	31	TAGAAT
		SMSK321_0337	CttT**TT**GAa	12	G**CA**A**TT**CA**G**	31	TAGAAT
	*sigX1*	SP0014	G**CA**G**TT**TA**G**	12	A**CA**GaaTG**G**	32	TAGACT
		SM12261_0014	G**CA**G**TT**GA**G**	12	A**CA**GaaTA**G**	32	TAGACT
		SMSK321_1651	G**CA**G**TT**TA**G**	12	A**CA**GaaTG**G**	32	TAAACT
	*sigX2*	SP2006	G**CA**G**TT**TA**G**	12	A**CA**GaaTG**G**	32	TAGACT
		SM12261_0613	G**CA**G**TT**TA**G**	12	A**CA**GaaTG**G**	31	TAAACT
		SMSK321_1251	G**CA**G**TT**TA**G**	12	A**CA**GaaTG**G**	32	TAGACT
Fratricide immunity	*comM*	SP1945	A**CA**T**TT**GA**G**	10	A**CA**G**TT**CTC	13	TATAAT
		SM12261_0911	G**CA**T**TT**TA**G**	12	A**CA**G**TT**GA**G**	32	TATAAT
		SMSK321_1653	G**CA**T**TT**TA**G**	12	A**CA**G**TT**GA**G**	32	TATAAT
Unknown functions	*ribF*	SP1110	A**CA**C**TT**CAt	12	A**CA**T**TT**CA**G**	27	TATGAT
		SM12261_0833	A**CA**C**TT**CA**G**	12	A**CA**T**TT**CA**G**	27	TATGAT
		SMSK321_1087	T**CA**G**TT**CA**G**	12	A**CA**T**TT**GG**G**	27	TATGAT
	Peptide deformylase	SP1549	A**CA**G**TT**GA**G**	11	G**CA**G**TT**ATc	20	TATAAT
		SM12261_1162	A**CA**G**TT**TA**G**	11	G**CA**G**TT**ATc	20	TATAAT
		SMSK321_0137	A**CA**G**TT**GA**G**	11	G**CA**G**TT**GCc	20	TATAAT
	ABC transporter	SP1717	A**CA**A**TT**CA**G**	12	A**CA**G**TT**GA**G**	31	TATAAT
		SM12261_0940	A**CA**A**TT**CA**G**	12	A**CA**G**TT**GA**G**	31	TATAAT
		SMSK321_1696	A**CA**A**TT**CA**G**	12	A**CA**G**TT**GA**G**	31	TATAAT
	SPFH domain	SP2156	A**CA**A**TT**CAc	12	A**CA**T**TT**CA**G**	42	TATAAT
		SMSK321_1581	A**CA**A**TT**CAc	12	A**CA**T**TT**CA**G**	42	TATAAT
	Hypoth. protein	SP0429	–	–	CCAGTTGAG	32	TATACT
		SMSK321_0027	A**CA**C**TT**CA**G**	12	A**CA**G**TT**GA**G**	31	TATACT

Bases matching the direct repeat consensus for *S. pneumoniae* are highlighted in bold [8]. Bases divergent from the consensus are represented by lower case letters. A TIGR4 orthologue and its ComE-box sequence is described for each *S. mitis* gene

^a^Gene number from HOMD

^b^Consensus sequence in pneumococcus according to [8]

### Late response: conservation of the SigX regulon

**Fig. 3:**
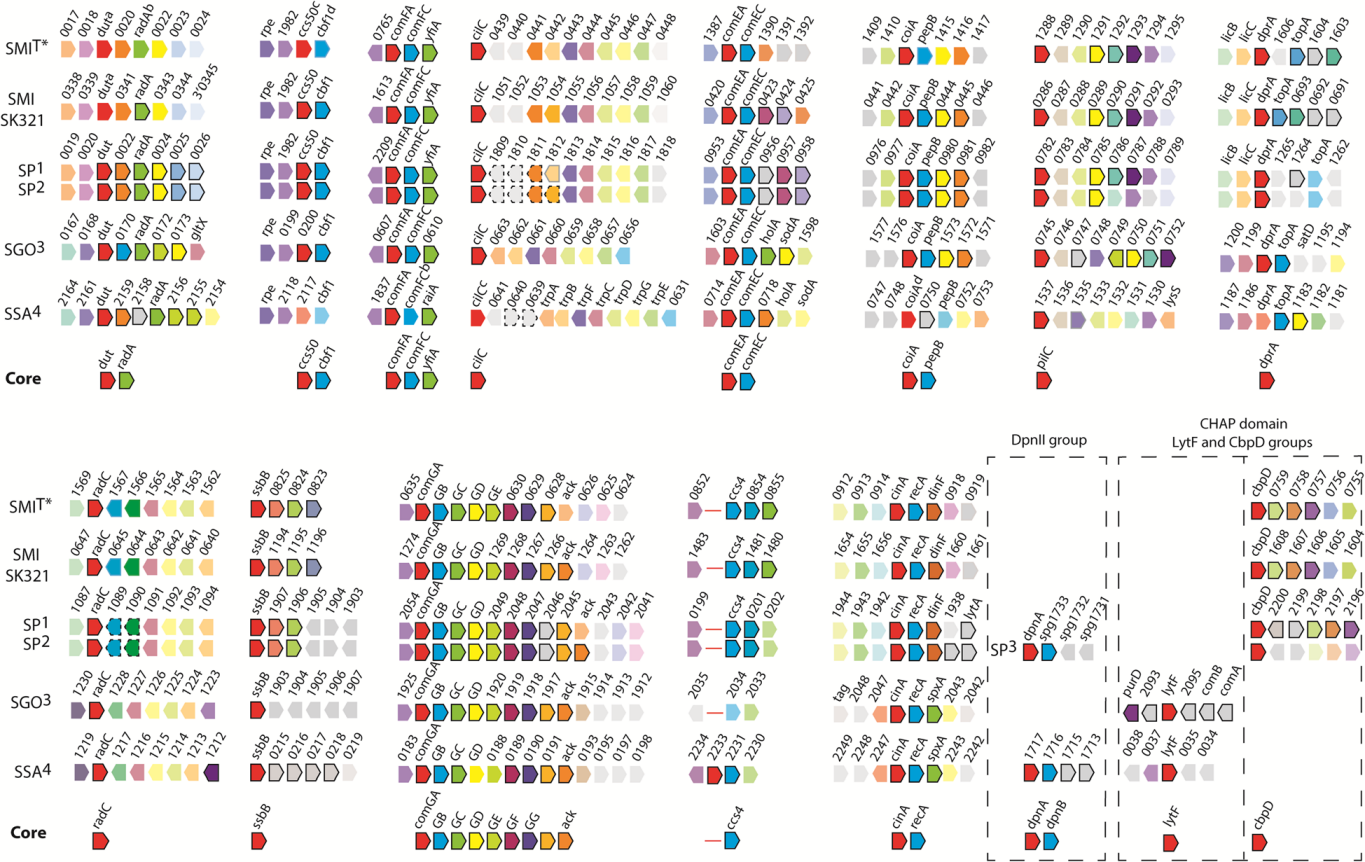
Core genes SigX regulon of the mitis group. Synteny conservation of *S. mitis* type strain and SK321, *S. pneumoniae*, *Streptococcus gordonii* and *S. sanguinis*. Red pentagons correspond to genes immediately downstream of a SigX box. Genes induced by competence, black borders; upregulated genes oriented antisense to the gene downstream of a SigX box, dashed black borders; no change in gene expression, borderless faded colors; orthologues in each of the 15 gene groups are represented by similar colors; no orthologues in the regions analyzed, gray pentagons. SMI^T*^, *S. mitis* type strain upregulated sequences in TSB and C + Y_YB_ media, gene locus tag as in GenBank (accession no. AEDX00000000); SK321 gene locus tag as in GenBank (accession no. AEDT00000000). SP^1^, *S. pneumoniae* Rx [9]; gene locus tag as in GenBank (*S. pneumoniae* TIGR4, accession no. AE005672). SP^2^, *S. pneumoniae* R6 [28]; gene locus tag as in GenBank (*S. pneumoniae* TIGR4, accession no. AE005672). SP^3^, *S. pneumoniae* G54 [50]; gene locus tag as in GenBank (S. pneumoniae G54, accession no. CP001015). SGO^3^, *S. gordonii* Challis [36]; gene locus tag as in GenBank (*S. gordonii* Challis, accession no. CP000725). SSA^4^, *S. sanguinis* SK36 [31]; gene locus tag as in GenBank (accession no. CP000387.1). ^a, b, c, d^ Upregulation was not > 2-fold. The image was modified from [29]

Late CSP-induced genes make the largest group of competence-specific products involved in binding, uptake, processing and integration of exogenous DNA, and the production of killing factors. Table 3 shows information on upregulated sequences in the two *S. mitis* strains and their orthologue late genes in *S. pneumoniae* [9]. Core genes, under regulation of SigX in the majority of competent streptococcal species [29] are in bold. Overall, upregulated genes were organized in 15 TARs (Fig. 3). For both strains, orthologues of late genes involved in DNA uptake were grouped in two significantly upregulated operons (*comGA*-*comGG* and *comEA-comEC*). Both operons are composed by genes required for assembly of the DNA transport and uptake machinery and are known as indispensable for transformation in *S. pneumoniae*. In addition, *comFA*, involved in DNA transport, presented a strong induction in both isolates. Four upregulated operons accounted for 5 DNA recombination genes*: ssbB* (NCTC12261^T^, 98.6; SK321, 697.9-fold), *dprA* (NCTC12261^T^, 63.8; SK321, 339.3-fold), *coiA* (NCTC12261^T^, 58.6; SK321, 139.6-fold), and *cinA-recA* (NCTC12261^T^, 21.7; SK321, 55.3-fold) and (NCTC12261^T^, 5.3; SK321, 18.8-fold), respectively. These genes are essential for the efficient replacement of donor DNA strands. As mentioned, fratricide has been demonstrated not only for pneumococcus, but also for commensal streptococci [30]. In the present analysis, the gene encoding the murein hydrolase essential for the pneumococcal fratricide mechanism, *cbpD,* was significantly upregulated (NCTC12261^T^, 116-fold; SK321, 458.5-fold). SP0031, a non-core late gene annotated as hypothetical protein in *S. pneumoniae* TIGR4, was also induced in both *S. mitis* isolates (non-annotated gene) (Table 3) and in our observations of *S. pneumoniae* D39 endogenous competence response (data not shown).

**Table 3:** *S. mitis* type strain and SK321 orthologues of *S. pneumoniae* late genes in response to CSP in C + Y_YB_

	Function in competence	*S. pneumoniae*		*S. mitis*				Description
		ID	Mean fold-change	ID		Mean fold-change^c^		
		TIGR4^b^	R6^d^	NCTC12261^a^	SK321^a^	NCTC12261	SK321	
SigX Core Orthologues [29]	DNA uptake and recombination	0023	10	0021	0342	1.4	2.3	DNA repair protein RadA; *radA*
		0978	16	1411	01508a^f^	58.6	139.6	Competence protein; *coiA*
		1266	64	1607	0695	63.8	339.3	DNA protecting protein DprA; *dprA*
		1908	64	0826	1193	98.6	697.9	Single-strand binding protein family; *ssbB*
		1940	16	0916	1658	5.3	18.8	Protein RecA; *recA*
		2208	128	0765	1614	113.2	591.7	Competence protein ComFA; *comFA*
		2207	64	01089^f^	00402^f^	133.6	1167.9	Competence protein, putative; *comFC*
		1808	16	0438	1050	57.0	694.6	Type 4 prepilin peptidase; *cilC*, *pilD*
		0954	32	1388	0421	122.8	627.9	ComE operon protein 1; *comEA*
		0955	64	1389	0422	93.6	839.4	DNA internalization-related competence protein ComEC
		2047	64	0629	1267	78.3	218.4	Methyltransferase small domain superfamily
		2048	64	0630	1268	187.6	992.0	ComG operon protein 6
		2049	64	01214^f^	1269	141.4	1016.1	Hypothetical protein; *cglE*
		2050	64	0631	1270	164.6	852.6	Competence protein
		2051	64	0632	1271	192.2	1027.7	Competence protein
		2052	64	0633	1272	162.5	1079.0	Competence protein CglB; *comYB*
		2053	64	0634	1273	146.1	929.8	Putative ABC transporter subunit ComYA; *comYA*
	Lysis	2201	64	0760	1609	134.5	458.5	Choline binding protein D; *cbpD*
	Unknown functions in competence	0021	10	0019	0340	1.2	1.6	Deoxyuridine 5′-triphosphate nucleotidohydrolase, *dut*
		1981	8	0563	1204	1.3	4.3	Competence-induced protein Ccs50; *ccs50*
		1980	8	01275^f^	00562^f^	1.5	3.9	CMP-binding factor; *yhaM*, *cbf1*
		2206	32	0764	1613	2.2	4.1	Ribosome-associated factor Y; *yfiA*
		0979	8	1412	0443	1.5	3.4	Oligopeptidase F; *pepB*
		0782	8	1288	0286	7.1	47.2	Conserved domain protein; *pilC*
		1088	32	1568	0646	167.3	297.6	DNA repair protein RadC; *radC*
		2044	-^e^	0627	1265	1.6	4.5	Acetate kinase; *ackA*
		1941	32	0915	1657	21.7	55.3	Competence/damage-inducible protein CinA; *cinA*
Non-Core Orthologues	Unknown functions in competence	0024	8	0022	0343	1.4	2.4	Carbonic anhydrase
		0025	4	0023	0344	1.3	2.2	Membrane protein, putative
		0031	16	00941^f^	0345	6.7	1.2	Hypothetical protein
		0030	32	0025	0346	6.6	26.1	Competence-induced protein; Ccs16
		0029	4	0026	0347	5.0	1.9	Conserved hypothetical protein
		2045	8	0628	1266	3.3	19.8	Adenine-specific methyltransferase
		2197	16	0757	1606	3.7	86.4	ABC transporter substrate-binding protein
		2198	16	0758	1607	3.0	118.7	ABC transporter, permease protein
		2199	4	0759	1608	3.7	92.9	Conserved hypothetical protein
		1939	16	0917	1659	4.4	10.8	DNA-damage-inducible protein
		0980	6	1413	0444	1.7	3.9	O-methyltransferase family protein
		0981	4	1414	0445	1.3	2.7	Foldase protein PrsA
		1072	8	1421	0451	1.4	2.7	DNA primase
		1073	8	1422	0452	1.3	3.1	RNA polymerase sigma factor RpoD
		1074	8	1423	0453	1.3	3.0	N-6 Adenine-specific DNA methylase YitW
		0201	8	0853	1482	3.2	13.5	Competence-induced protein Ccs4; *ccs4*

^a^Gene number and product from HOMD

^b^Gene number and product from [51]

^c^Mean fold-change induction of CSP in S. *mitis* type strain and SK321 obtained by transcriptome analysis

^d^Expression pattern during response the of *S. pneumoniae* to CSP pheromone [9]

^e^No upregulation detected by [9]

^f^Gene number according to PROKKA annotation [52]

**Fig. 4:**
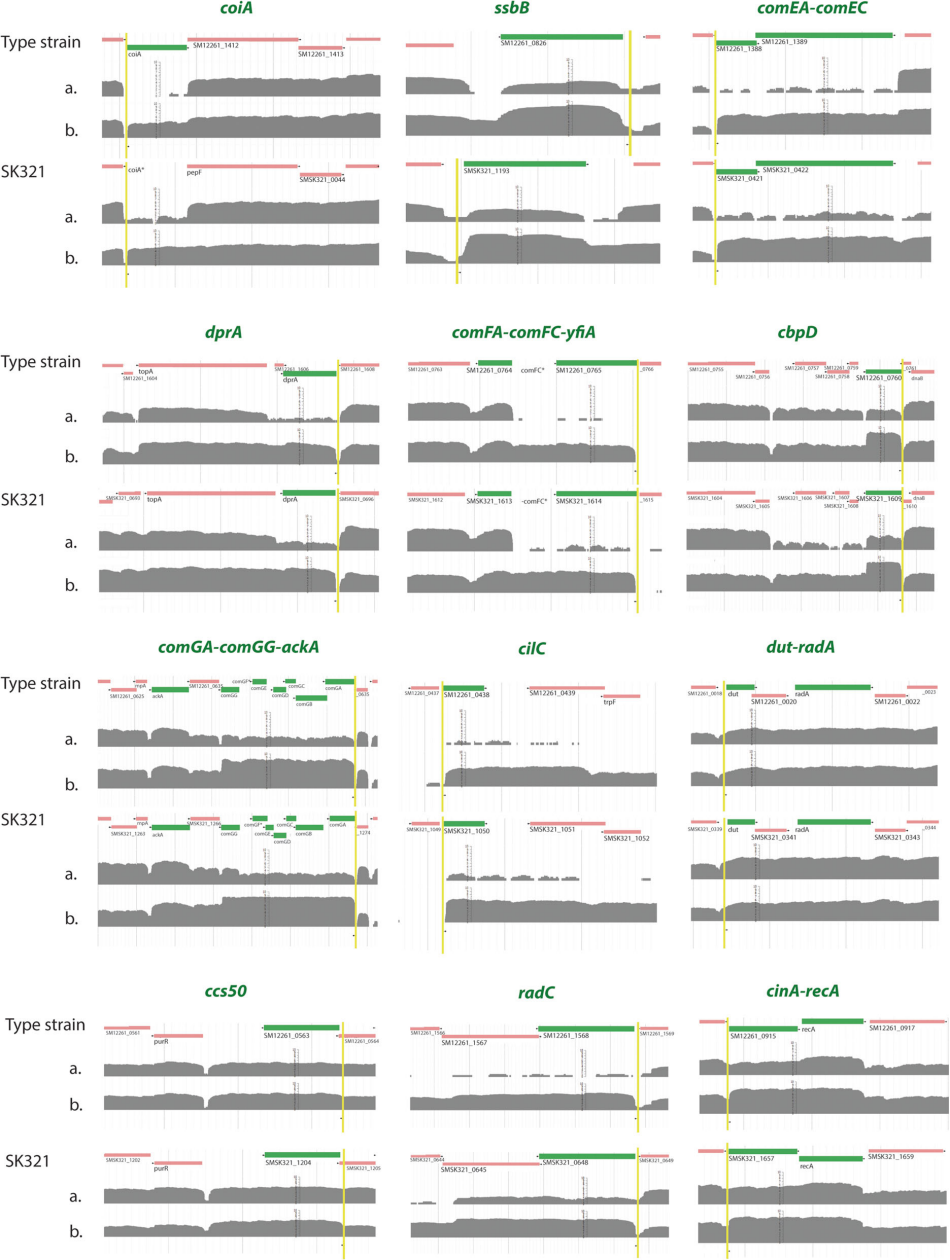
Similarity of transcriptome profiles in the transcriptional initiation regions of SigX regulon core genes [29] in the type strain and SK321. The SigX core genes are marked in green. The arrows highlighted in yellow show the region where sequences matching the SigX-box consensus were found. Line a. corresponds to the control culture and line b. to the culture treated with CSP. Comparison between lines a. and b. shows the higher expression found in samples treated with the pheromone. Stars followed by gene annotation represent non-annotated sequences in *S. mitis* genomes

SigX, together with RNAP, recognizes a “cinbox” or “SigX-box” unique DNA element (− 10 from the transcription start and featuring a T-rich region at − 25) in the promoter regions of late genes, initiating their transcription [7]. To search for conserved promoter elements in *S. mitis*, the regions immediately upstream of the potential start sites of orthologues of *S. pneumoniae* late operons were aligned (Fig. 4). Table 4 displays SigX-box sequences with no more than one mismatch detected upstream of 13 SigX core genes; combined with upregulation of downstream genes, these account for a total of 26 SigX controlled genes. Among the nine SigX core genes with unknown functions in competence, eight were upregulated at least 2-fold in SK321 (*ccs50, cbf1, yfiA, pepB, pilC, radC, ackA, cinA*), and four in the type strain (*yfiA, pilC, radC, cinA*). In addition, a non-annotated gene upstream of *ccs4* (SM12261_0853; SMSK321_1482) coding for a 46-aa long peptide was upregulated in both *S. mitis* strains. Although orthologues of this gene are upregulated during competence in *S. sanguinis* (gene SSA_2233) [31], *S. mutans* UA159 (gene SMU.2076) [29] and *S. pneumoniae* R6 (gene SPR_0181 annotated as *orf47*) [28], this peptide remains uncharacterized and its role in competence is still unknown. We identified five additional *S. mitis* genes with candidate sites matching the SigX-box consensus – but without orthologues in *S. pneumoniae* - (Table 4), which we discuss further below.

**Table 4:** Alignment of DNA sequences found upstream of *S. mitis* type strain and SK321 putative late genes. Distances to the start codon of first ORF are shown

	Function in competence	Annotation	Gene ID	Consensus				
				TTT	0	TACGAATA		
SigX Core Orthologues [29]	DNA recombination	*coiA*	SM12261_1411	TTTTT	−0-	**TACGAATA**	−22-	ATG
			SMSK321_na	TTTTT	−0-	**TACGAATA**	−21-	ATG
		*dprA*	SM12261_1607	TTTTTT	−0-	**TACGAATA**	−21-	ATG
			SMSK321_0695	TTTTT	−0-	**TACGAATA**	−21-	ATG
		*ssbB*	SM12231_0826	TTTT	−1-	**T**g**CGAATA**	−25-	ATG
			SMSK321_1193	TTT	−2-	**TACGAATA**	−20-	ATG
		*comGA*	SM12261_0634	TTTTT	−9-	**T**g**CGAATA**	−25-	ATG
			SMSK321_1273	TTTTT	−9-	**T**g**CGAATA**	−20-	ATG
	DNA uptake	*comFA*	SM12261_0765	TTTTT	−9-	**TACGAATA**	−25-	ATG
			SMSK321_1614	TTTT	−0-	**TACGAATA**	−7-	ATG
		*cilC*	SM12261_0438	TTTTT	−0-	**TACGAATA**	−7-	ATG
			SMSK321_1050	TTTTT	−0-	**TACGAATA**	−7-	ATG
		*comEA*	SM12261_1388	TTTTTT	−8-	**TACGAATA**	−19-	ATG
			SMSK321_0421	TTTTTT	−8-	**TACGAATA**	−19-	ATG
	Fratricide	*cbpD*	SM12261_0760	TTTTTTT	−1-	**T**c**CGAATA**	−57-	ATG
			SMSK321_1609	TTTTTTT	−1-	**T**c**CGAATA**	−49-	ATG
	Unknown functions	*dut*	SM12261_0019	TTTT	−0-	**T**c**CGAATA**	−48-	ATG
			SMSK321_0340	TTTT	−0-	**T**c**CGAATA**	−48-	ATG
		*ccs50*	SM12261_0563	TTT	−18-	a**ACGAATA**	−74-	ATG
			SMSK321_1204	TTT	−18-	a**ACGAATA**	−74-	ATG
		*pilC*	SM12261_1288	TTT	−0-	**TACGAATA**	−30-	ATG
			SMSK321_0286	TTT	−0-	**TACGAATA**	−30-	ATG
		*radC*	SM12261_1568	TTTTT	−9-	c**ACGAATA**	−20-	ATG
			SMSK321_0646	TTTT	−10-	c**ACGAATA**	−20-	ATG
		*cinA*	SM12261_0915	TTTTTT	−8-	**TACGAATA**	−17-	ATG
			SMSK321_1657	TTTTTT	−8-	**TACGAATA**	−17-	ATG
Non-Core Orthologues	Unknown function	5′ *ccs4*	SM12261_5’0853	TTT	−0-	**TACGAATA**	−19-	ATG
			SMSK321_5’1482	TTT	−0-	**TACGAATA**	−19-	ATG
		*ccs 16*	SM12261_5’0025	TTT	−0-	**T**c**CGAATA**	−68-	ATG
			SMSK321_5’0346	TTT	−0-	**T**c**CGAATA**	−14-	GTG
Non-Core Without Orthologues	Putative bacteriocin	Cib-like operon	SM12261_0044	TTT	−0-	**T**t**CGAATA**	−33-	ATG
			SMSK321_1305	TTTTT	−0-	**T**t**CGAATA**	− 229	ATG
	Unknown function	Lipoprotein	SM12261_0750	TTTT	−0-	**TACGAATA**	−147	ATG
			SMSK321_1599	TTTT	−2-	**TACGAATA**	−147	ATG
		Hypot. protein	SMSK321_1184	TTTT	−12-	a**ACGAATA**	−13-	GTG

Bases in agreement to cinbox (SigX-box) consensus in bold [33]. Bases divergent from the consensus are represented by lower case letters. Only the first genes within each induced transcriptionally active region are shown

### Downregulated genes during competence development

Genes downregulated during the state of competence in the mitis group are fewer in number than upregulated sequences, and still not well characterized anywhere. SK321 isolate presented 70 genes with a 2- to 10-fold decrease in gene expression (Additional file 3: Table S3), which mostly included genes involved in sugar and amino acid metabolism, alcohol dehydrogenases, and hypothetical proteins, and 10 orthologues of previously reported *S. pneumoniae* CSP-repressed genes [9]. *S. mitis* type strain had fewer downregulated sequences, independently of the medium used (Additional file 1: Table S1 and Additional file 2: Table S2). Only two genes were commonly repressed in TSB and C + Y_YB_ cultured cells, both uncharacterized hypothetical proteins, and the difference may be explained by the different growth conditions. Among the other downregulated sequences in the type-strain, none were orthologues of genes reported at least once as being repressed by CSP in any other streptococci. This is not an isolated observation, since a previous transcriptome analysis of *S. pneumoniae* competent cells has shown strain-specific responses also for downregulated genes [28]. Furthermore, the downregulation of these genes can also be an indirect effect of a general stress response caused by CSP.

### Identification of early and late genes in *S. mitis* with no orthologues in *S. pneumoniae*

**Fig. 5:**
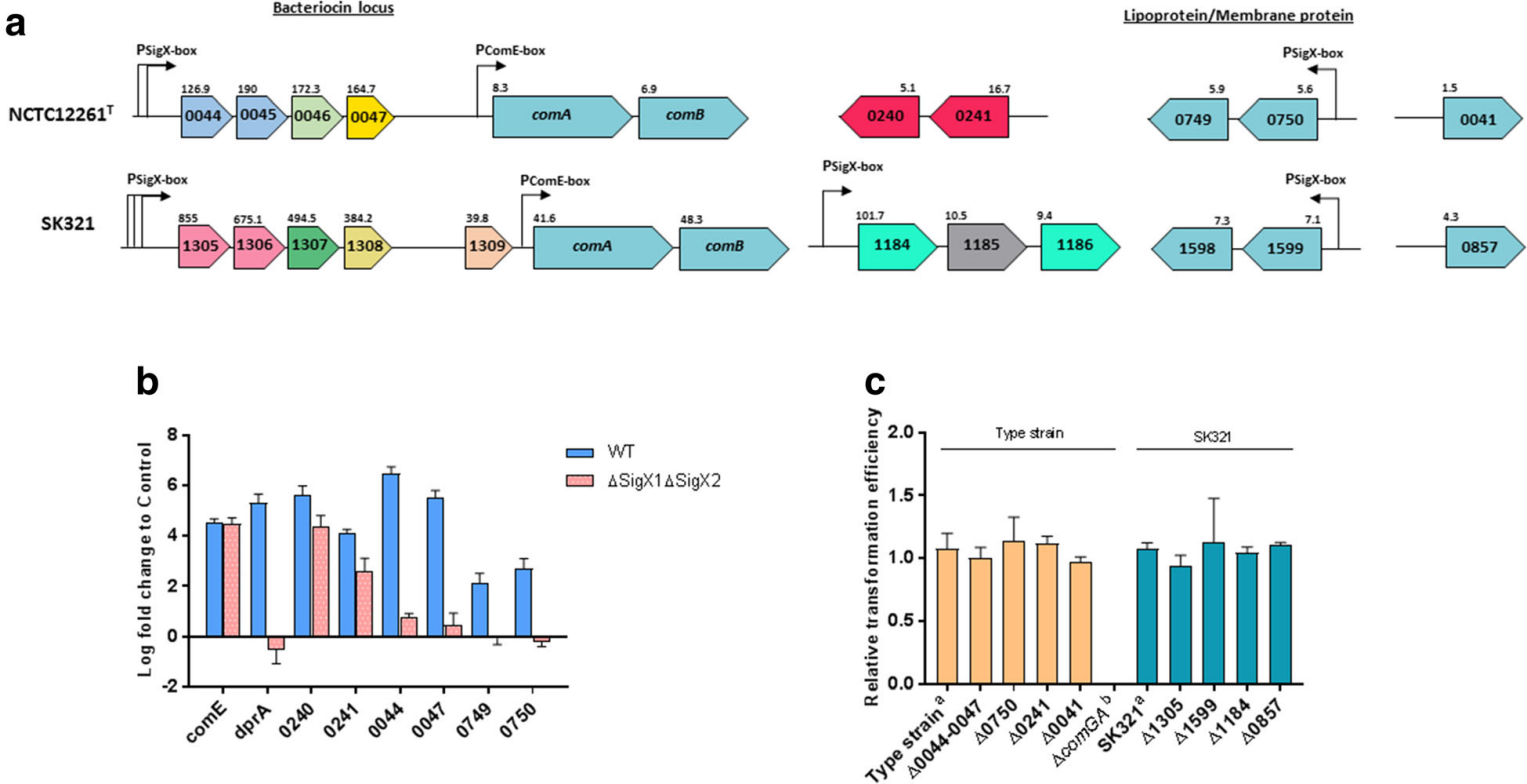
**a** Identification of early and late CSP-induced genes in *S. mitis* with no orthologues in *S. pneumoniae*. Presence and number of putative promoter sites are represented by black arrows. Fold-change induction values are presented as numbers on top of the pentagons. Orthologue genes are represented by the same color in both strains. **b** Real-Time PCR results regarding the expression of *S. mitis* type strain genes after the treatment with the pheromone compared to the untreated culture in a *ΔsigX1ΔsigX2* derivative of the same strain (dashed bars) or the wild-type (filled bars). Controls for early genes were *comA* and *comE*; control for late gene was *dprA*. **c** Relative transformation efficiency of the *S. mitis* knockout strains for the competence-induced genes. ^a^ Positive control; ^b^ Negative control

Despite the close genetic kinship between these species, some *S. mitis* upregulated genes lack orthologues in *S. pneumoniae* strains (Fig. 5a, Additional file 4: Table S4). Four to five small adjacent ORFs located upstream the ABC transporter ComAB were found induced by CSP in both *S. mitis* strains. SM12261_0044–0045 and SMSK321_1305–1306 encode peptides with a GG-type leader sequence and a bacteriocin moiety featuring a GxxxG-like motif [32]. The processing of SM12261_0044–0045 at the Gly-Gly site would give mature peptides of 20 and 32 amino acids residues, while SMSK321_1305–1306 processing would result in 40- and 33- amino-acids mature peptides, respectively. Interestingly, in silico analysis revealed no similarity between active peptide sequences of the two strains. However, the leader sequence is identical for the peptides encoded by SM12261_0045 and SMSK321_1305, which suggests they might be processed and exported by the same bacteriocin transporter. The third gene in each operon codes for another GG-leader peptide, and may play a role as a signaling peptide involved in bacteriocin production. Finally, based on the typical transcriptional pattern of class IIb bacteriocins, the fourth gene possibly codes for an immunity protein. In the promoter region of SM12261_0044 and SMSK321_1305, we identified an usual sequence (TTCGAATA) that matches the SigX box consensus, suggesting that these genes are regulated by SigX (Fig. 5a) [7, 33]. We confirmed this prediction by RT-PCR in a *S. mitis* strain lacking the two copies of *sigX* (Fig. 5b). Thus, this operon appears to be involved in the production and export of competence-related bacteriocins and part of the late CSP response in *S. mitis*.

Both strains possess upregulated operon (SM12261_0749–0750; SMSK321_1598–1599) that codes for a membrane protein and a lipoprotein, respectively. We identified orthologues of SM12261_0750 and SMSK321_1599 in several *S. sanguinis* strains, in addition to a dozen strains of *S. mitis*, while orthologues of SM12261_0749 and SMSK321_1598 were only detectable in other *S. mitis* strains. In *S. sanguinis* SK36 (SSA_2192), this gene is upregulated during competence, and a SigX-box site marks its promoter region [31]. We found the same SigX-box sequence upstream of SM12261_0750 and SMSK321_1599 (Fig. 5a), and confirmed its activity through RT-PCR (Fig. 5b).

Another region composed by two ORFs coding for two hypothetical proteins was upregulated by CSP in both media tested, but only in the type strain. BLAST analyses revealed orthologue sequences of SM12261_0241 in other three *S. mitis* strains (SK1073_0114, SK569_0006 and SK616_1612), and the gene annotation corresponds to a bacteriocin class II with double glycine leader peptide in all of them. Interestingly, the active part of the peptide encoded by SM12261_0241 is identical to the one encoded by isolate SK569. In turn, gene SM12261_0240 is annotated as a conserved hypothetical protein, but its orthologues in SK616_1615, SK569_0169 and SK579_0521 code for Enterocin A immunity proteins. We were not able to identify either ComE-box or SigX-box sequences in the promoter region of SM12261_0241. However, analysis of SM12261_0240-SM12261_0241 mRNA expression in a *ΔsigX1ΔsigX2* mutant showed upregulation of this transcript (Fig. 5b), confirming its independence from the alternative sigma factor regulation. Indeed, this suggests that this region might be suffering indirect ComE regulation or may simply reflect a broader specificity of ComE than previously thought.

Gene SMSK321_1184, coding for a hypothetical protein, was upregulated more than 100-fold in SK321 but has no orthologue in the type strain. Using BLAST analyses against streptococcal genomes, three orthologues of this gene with at least 70% identity were identified in *S. mitis* SK1073, *Streptococcus salivarius* 57.I and *S. sanguinis* SK1056. A putative SigX-box sequence with one mismatch in the first nucleotide was detected upstream of SMSK321_1184 (Table 4), as well as in the promoter regions of all three orthologues. In fact, the presence of this gene in three different species might indicate the importance of this protein in competence throughout the evolution of salivarius and mitis groups of streptococci.

Figure 5c demonstrates that deletion of these unique CSP-responsive genes of *S. mitis* type strain and SK321 did not affect transformation yields in any of the knockout strains. Thus, although regulated by CSP, these genes seem dispensable for DNA uptake and processing in these strains.

## Discussion

Commensal streptococci are pioneer colonizers of the oral cavity that attach to the tooth surfaces, and to which more pathogenic bacteria later adhere to establish a mature multispecies biofilm. This close attachment provides a genetic pool to competent oral streptococci, which become potential recipients for horizontally transferred DNA as well as latent reservoirs of important genetic elements, such as antibiotic resistance genes [15]. More importantly, this attribute may also increase the likelihood of survival for some members of the population during stress conditions. To date, the global transcriptome responses during competence have  been studied in only three oral streptococci, *S. mutans* [29, 34, 35], *S. sanguinis* [31] and *S. gordonii* [36], and despite a few reports of competence in *S. mitis* [22, 30, 37], little or nothing is known about its regulation or response specificity in different strains. Recently, sequence of the genomes of a range of *S. mitis* revealed that truncation in genes required for competence was a common feature, present in roughly 40% of the strains [20]. However, even when apparently possessing a complete intact competence apparatus, only a few of the remaining strains displayed transformability under laboratory conditions [22]. Interestingly, the *sigX* is apparently the most common affected gene (absence and/or truncation), while DNA uptake and bacteriocin genes are largely conserved throughout *S. mitis* strains. Our transcriptome data revealed that the overall response of two competent *S. mitis* oral isolates to CSP resulted in significant upregulation of genes involved in the modulation of the competence cascade, DNA uptake and recombination, as well as lysis and bacteriocin production. Furthermore, we identified unique upregulated sequences, a fact that highlights the *S. mitis* singularity despite its close genetic resemblance to *S. pneumoniae*.

In the present study, the comparison between two *S. mitis* strains located in different evolutionary branches provided insight into the variation of the global transcriptomes of isolates of the same species under the same growth conditions. When cultured in the competence permissive medium C + Y_YB_, approximately 4% of the type strain genome positively responded to CSP, compared to almost 10% in SK321. All orthologues of ComE-responsive genes were upregulated at least 2-fold in SK321, while the type strain displayed a weaker response mainly for *comW* and *comCDE* (Table 1). However, this difference might be simply due to a smaller amplification of the CSP signal in this strain, since their transcriptomic maps show immediate expression downstream of their ComE binding sites. A recent study comprising transcriptomic data from at least five species from different phylogenetic groups, showed that streptococci regulate a core of 27 to 30 pan genes under the control of the alternative sigma factor SigX [29]. In the present analysis, the 12 SigX core genes required for DNA uptake in *S. pneumoniae* were upregulated in both strains, as well as the six genes required for DNA recombination (Table 3 and Fig. 3). Additionally, the SigX regulon comprises genes involved in lytic attack, and the key protein in fratricide, CbpD was strongly upregulated in both *S. mitis* isolates. Not surprisingly, among the orthologues of early genes and preceded by a ComE binding site was also *comM*, strongly implying that *S. mitis* employs a similar immunity mechanism to CbpD as does *S. pneumoniae*. 

CSP-regulated bacteriocin production is a common feature among naturally competent streptococci. *S. mutans*, one of the most distinguished bacteriocin producers, coordinates the CSP-ComDE regulation with production of mutacins [38], while *S. pneumoniae* and *S. gordonii* carry a direct link between SigX regulation and bacteriocin production [39, 40]. The most striking difference when comparing *S. mitis* orthologues of *S. pneumoniae* late genes was the absence of the *cibABC* bacteriocin locus in the two strains studied. Previous reports have shown that some *S. mitis* strains did not maintain this competence-induced locus throughout their evolution from *S. pneumoniae*, and that they probably acquired other genes associated with the production of killing factors [41, 42]. Indeed, we identified a strongly upregulated bacteriocin locus in a single transcriptional unit located upstream of the *comAB* operon, with a conserved link to the late competence response by carrying two (in the type strain) or even three (in SK321) copies of the SigX box in their promoter regions (Fig. 5b). Although there are no previous reports, nor clear explanations for the role of multiple SigX box sequences, we hypothesize that recurrent recombination events in this region might have either left or removed additional promoter sequences. Puzzlingly, in *S. pneumoniae* TIGR4, this region is occupied by the bacteriocin encoding gene *blpU* preceded by a Blp-box, while in *S. pneumoniae* D39 there is also a transposase gene transcribed in the opposite direction [41]. This suggests that a shuffling between Blp and competence-induced bacteriocins might have occurred during the parallel evolution of the two species.

Besides this bacteriocin operon, we also detected other upregulated sequences without orthologues in *S. pneumoniae*. These accounted for hypothetical proteins, membrane proteins, lipoproteins and even other bacteriocin-like encoding operon. In fact, by comparative analyses of *S. mitis* type strain and *S. pneumoniae* TIGR4, D39 and G54, Kilian et al. [20] showed that 100 *S. mitis* proteins do not present any homology with any *S. pneumoniae* strain, and that 83 of the 100 *S. mitis* proteins without homologues in *S. pneumoniae* had homologues in *S. sanguinis*, *S. gordonii*, *S. agalactiae* and *S. thermophilus* and none lacked homologues in other bacteria. While there is no information on whether there are competence-related proteins among these, the presence of *S. mitis* strain specific genes suggests that they were acquired more recently in evolution, independently by individual *S. mitis* lineages. 

## Conclusions

Data gathered throughout the last two decades have provided great understanding about the streptococcal regulation of competence in various groups of this genus, reinforcing the fact that competence for genetic transformation is a conserved trait among streptococci. Overall, our results demonstrated that in two *S. mitis* isolates CSP induces a global change in gene expression in two *S. mitis* isolates that not only supports the maintenance of the competent state and the DNA uptake machinery, but also strongly induces expression of genes involved in lysis and bacteriocin production. Most of the previously described competence-induced loci in other streptococci were detected by our method together with several other novel genes, for which functions remain to be elucidated. Furthermore, promoter analysis of the genes not previously known to be induced during *S. mitis* competence suggests that several of them belong to either the ComE or the SigX regulons. These findings reveal conservation of the competence system in transformable *S. mitis* strains and highlight the characteristics of strain-specific regulated regions. Particularly, our findings are significant not only from a fundamental understanding of competence in streptococci, but also from a practical perspective, as transformation is an important tool to explore gene functions and to design *S. mitis* for potential applications such as vaccine development.

## Methods

### Bacterial strains and growth conditions

All bacterial strains and isogenic derivatives used in this study are listed in Additional file 5: Table S5. Bacterial stocks were stored at − 80 °C in Todd Hewitt Broth (THB, Becton Dickinson and Company, Le Pont de Claix, France) or Tryptic Soy Broth (TSB, Soybean-Casein Digest medium, BactoTM) supplemented with 30% glycerol. Pre-cultures were prepared from fresh liquid cultures grown in TSB at 37 °C 5% CO_2_ until an absorbance of 0.5 at 600 nm (optical density at 600 nm [OD_600_]; Biophotometer; Eppendorf), supplemented with 15% glycerol and stored at − 80 °C. For transformation, RT-PCR and RNA sequencing assays, C + Y_YB_ medium [43] assembled as described previously [44] was used.

### Synthetic peptides

Lyophilized CSPs of *S. mitis* NCTC12261^T^ (NH_2_- EIRQTHNIFFNFFKRR-COOH) and SK321 (NH_2_-ESRLPKIRFDFIFPRKK-COOH) were synthesized by GenScript (GenScript Corporation, NJ), with purity > 95%. Stock solutions were prepared by re-suspending the material in distilled water to a concentration of 10 mM, and stored at − 20 °C. Working stocks at a 100 μM concentration were aliquoted and stored at − 20 °C.

### Transformation

Transformation kinetics was carried out as previously described [22]. Briefly, pre-cultures of NCTC12261^T^ and SK321 were diluted 100-fold in C + Y_YB_ and grown at 37 °C, 5% CO_2_ until an OD_600_ of 0.04 was reached. After the addition of 300 nM of CSP, 1 μg ml^− 1^ recombinant plasmid pVA838 was added at various time points. Cells were incubated at 37 °C for 30 min before addition of 20 U ml^− 1^ DNaseI (Roche, DNaseI recombinant, 10 U ml^− 1^), followed by incubation at 37 °C for further 40 min to remove extracellular DNA. Transformants were selected on blood agar plates supplemented with Erythromycin by 24 h of incubation at 37 °C, 5% CO_2_.

### Construction of mutants

For construction of the *S. mitis* Δ*sigX1*Δ*sigX2* (MI014), two techniques were used. First, the standard PCR ligation mutagenesis strategy was employed [45], with minor modifications, to delete *sigX1*. Briefly, the *sigX1* flanking regions were amplified using primer pairs FP395–FP396 and FP397–FP398. The kanamycin resistance cassette (Km^R^) was amplified using the primer pairs FP001–FP068. Further, ligation and purification of the PCR products were performed using T4 DNA ligase (Fermentas) and the QIAquick PCR purification kit (Qiagen), respectively. The final product was transformed into *S. mitis* NCTC12261^T^. The specific insertional inactivation of *sigX2* was performed with a recombinant integrational vector, pSF152 [46]. An internal fragment of *sigX2* was amplified by primer pair FP451-FP452 and was forced-cloned via *Bam*HI and *Eco*RI 5′ tags of the respective primers, into the corresponding sites of plasmid pSF152. The final products were transformed into the parent strains as previously described [22]. All primers used for mutant constructions are listed in Additional file 5: Table S5.

### Real time PCR

Pre-cultures of MI014 were diluted 100-fold in C + Y_YB_ to a final colume of 10 ml and incubated at 37 °C 5% CO_2_ until an OD_600_ of 0.04 was reached. The cultures were then divided in two, with one half being treated with 150 nM synthetic CSP and the other half kept untreated. Cultures were incubated for 15 min and pellets were harvested at 8000 g, 4 °C for 10 min. Total RNA was extracted using the High Pure Isolation Kit (Roche, Mannhelm, Germany) and treated with Turbo DNase (AM2238, Ambion, Life Technologies, Carlsbad, California, USA) to clear any DNA contamination. Complementary DNA templates were prepared from RNA First Strand cDNA Synthesis Kit (Thermo Fisher Scientific, Fermentas) according to manufacture’s protocol. Housekeeping *gyrA* gene was used to validate the results. The primers for the studied genes were designed by using Primer3Web Platform for uniformity in size (80-150 bp) and melting temperature. The primers sequences are provided in Additional file 4: Table S4. PCR conditions included an initial denaturation at 95 °C for 10 min, followed by a 40-cycle amplification consisting of denaturation at 95 °C for 30 s and annealing and extension at 55 °C for 1 min. Data were collected and analyzed with the software MxPro (Stratagene).

### RNA sequencing

Pre-cultures of NCTC12261^T^ and SK321 at OD_600_ of 0.5 were centrifuged at 8000 g, 4 °C, for 10 min, resuspendend 100-fold in TSB or C + Y_YB_ and the diluted cultures were incubated at 37 °C 5% CO_2_ until an OD_600_ of 0.04 was reached. Then, the total volume of culture was divided in two tubes and grown in the presence or absence of 150 nM of CSP. Following, cells were harvested for RNA extraction at 10000 g, 4 °C, for 10 min. Procedures for RNA extraction, RNA enrichment and preparation of DNA library for sequencing using Illumina® HiSeq were carried out as described elsewhere [47]. Following sequence run, a FASTQ file was derived from each sample. For differential expression analysis of genes, raw read counts for the *S. mitis* type strain and SK321 transcripts were generated using a Perl script based on the mapped read profiles of the two strains, as previously described [48]. The “DESeq” Bioconductor software package [49] was used for assessment of differential expression levels when comparing samples. In total, 16 transcriptomes were included in the study. The results are derived as means of 12 samples grown in the presence or absence of CSP in three independent biological experiments of *S. mitis* type strain and SK321 grown in C + Y_YB_. For growth in TSB, the results are derived as means of four samples collected in two independently conducted biological experiments. Genes that exhibited > 2-fold increase in mean read (with a *p* value < 0.05) were considered differentially expressed. Transcriptome maps were visualized using Jbrowser at http://bioinformatics.forsyth.org/mtd/.

## Additional files

Additional file 1: **Table S1.** Type strain upregulated genes (> 2-fold) in response to CSP in TSB [51, 52]. (DOCX 18 kb)

Additional file 2: **Table S2.** Type strain genes up and downregulated (> 2-fold) in response to CSP in C + Y_YB_. (DOCX 19 kb)

Additional file 3: **Table S3.** SK321 genes upregulated (> 2-fold) in response to CSP in C + Y_YB_. (DOCX 33 kb)

Additional file 4: **Table S4.** CSP responses by the type strain and SK321 genes without orthologues in *S. pneumoniae.* (DOCX 13 kb)

Additional file 5: **Table S5.** Strains, mutants and primers used in this study. (DOCX 19 kb)

## Abbreviations

CSP: Competence-stimulating peptide
DR: Direct repeat
GG: Glycine-glycine
HOMD: Human oral microbiome database
OD: Optical density
PCR: Polymerase chain reaction
RNAP: RNA polymerase
RNA-seq: RNA sequencing
RT-PCR: Reverse transcription polymerase chain reaction
TAR: Transcriptionally activated region
THB: Todd-Hewitt Broth
TSB: Tryptic Soy Broth
