# Supplementary material for: High-resolution profiles of the Streptococcus mitis CSP signaling pathway reveal core and strain-specific regulated genes
Source: BMC Genomics. 2018 Jun 13;19:453. doi: 10.1186/s12864-018-4802-y (PMC6001120; doi:10.1186/s12864-018-4802-y)
Supplement: Supplementary file 1 — Table S1. Type strain upregulated genes (> 2-fold) in response to CSP in TSB [51, 52]. (DOCX 18 kb) [file 12864_2018_4802_MOESM1_ESM.docx]

Additional file 1: **Table S1.** Type strain upregulated genes (>2-fold) in response to CSP in TSB.

| **Gene ID** | **Annotation** | | **Mean A**  **(MIWT-Control)** | **Mean B (MIWT-CSP)** | **Fold Change (B/A)** |
| --- | --- | --- | --- | --- | --- |
|  | |  |  |  |  |
| SM12261_0014 | competence-specific global transcription modulator | | 4092.35 | 40561.96 | 9.91 |
| SM12261_0016 | hypothetical protein | | 340.95 | 730.17 | 2.14 |
| SM12261_0017 | adenylosuccinate synthetase | | 196024.84 | 420981.44 | 2.15 |
| SM12261_0025 | competence-induced protein Ccs16 | | 58997.95 | 236854.87 | 4.01 |
| SM12261_0026 | hypothetical protein | | 16350.56 | 76208.87 | 4.66 |
| SM12261_0044 | hypothetical protein | | 451.15 | 104891.35 | 232.50 |
| SM12261_0045 | hypothetical protein | | 660.77 | 172172.32 | 260.56 |
| SM12261_0046 | bacteriocin-type signal sequence domain protein | | 998.51 | 194902.48 | 195.19 |
| SM12261_0047 | hypothetical protein | | 798.91 | 183972.32 | 230.28 |
| SM12261_0048 | ABC transporter CbaT | | 7411.02 | 1118067.25 | 150.87 |
| SM12261_0049 | transport protein ComB | | 8588.11 | 1193232.44 | 138.94 |
| SM12261_0056 | bifunctional purine biosynthesis protein PurH | | 3051.23 | 6821.03 | 2.24 |
| SM12261_0092 | hypothetical protein | | 1.04 | 75.23 | 72.14 |
| SM12261_0097 | Orf 10 protein | | 1.04 | 33.24 | 31.88 |
| SM12261_0105 | hypothetical protein | | 135.25 | 287.84 | 2.13 |
| SM12261_0240 | hypothetical protein | | 139.36 | 3400.50 | 24.40 |
| SM12261_0241 | hypothetical protein | | 506.57 | 10042.42 | 19.82 |
| SM12261_0438 | type 4 prepilin peptidase | | 271.67 | 24521.36 | 90.26 |
| SM12261_0591 | glutamate synthase small chain | | 1.04 | 38.55 | 36.97 |
| SM12261_0613 | competence-specific global transcription modulator | | 3244.66 | 27375.32 | 8.44 |
| SM12261_0629 | methyltransferase small domain superfamily | | 12885.13 | 534133.27 | 41.45 |
| SM12261_0630 | ComG operon protein 6 | | 2511.83 | 651908.61 | 259.54 |
| SM12261_0631 | competence protein | | 3610.90 | 931599.13 | 258.00 |
| SM12261_0632 | competence protein | | 1114.53 | 402618.42 | 361.24 |
| SM12261_0633 | competence protein CglB | | 4952.66 | 1070018.09 | 216.05 |
| SM12261_0634 | putative ABC transporter subunit ComYA | | 5124.17 | 1245404.18 | 243.05 |
| SM12261_0684 | hypothetical protein | | 41856.84 | 161180.67 | 3.85 |
| SM12261_0685 | caax amino protease family | | 55961.76 | 418443.11 | 7.48 |
| SM12261_0686 | hypothetical protein | | 38710.55 | 255531.73 | 6.60 |
| SM12261_0687 | hypothetical protein | | 20164.99 | 171104.76 | 8.49 |
| SM12261_0688 | hypothetical protein | | 9032.44 | 80070.70 | 8.86 |
| SM12261_0717 | immunity protein | | 96.27 | 539.76 | 5.61 |
| SM12261_0746 | M protein trans-acting positive regulator (MGA) | | 31116.09 | 86630.11 | 2.78 |
| SM12261_0749 | hypothetical protein | | 66421.94 | 408263.42 | 6.15 |
| SM12261_0750 | lipoprotein. putative | | 36726.70 | 454131.58 | 12.37 |
| SM12261_0760 | choline binding protein D | | 10765.69 | 1256272.41 | 116.69 |
| SM12261_0765 | competence protein ComFA | | 1576.86 | 96666.69 | 61.30 |
| SM12261_0787 | ComD | | 7559.53 | 285618.88 | 37.78 |
| SM12261_0788 | ComC | | 152.42 | 460.69 | 3.02 |
| SM12261_0826 | single-strand binding protein family | | 3862.04 | 477109.02 | 123.54 |
| SM12261_0833 | riboflavin biosynthesis protein RibF | | 103970.80 | 304164.30 | 2.93 |
| SM12261_0911 | lipoprotein. putative | | 1029.74 | 24167.85 | 23.47 |
| SM12261_0912 | hypothetical protein | | 26586.34 | 60510.61 | 2.28 |
| SM12261_0915 | competence/damage-inducible protein CinA N- domain. putative | | 86021.34 | 972821.61 | 11.31 |
| SM12261_0916 | protein RecA | | 268030.85 | 967119.29 | 3.61 |
| SM12261_0917 | DNA-damage-inducible protein | | 30632.46 | 84106.76 | 2.75 |
| SM12261_0940 | ABC transporter ATP-binding protein - Na+ export | | 42628.55 | 721682.55 | 16.93 |
| SM12261_0941 | ABC transporter membrane-spanning permease - Na+ export | | 45552.09 | 728264.11 | 15.99 |
| SM12261_0946 | hypothetical protein | | 29.59 | 293.33 | 9.91 |
| SM12261_0947 | gp19 | | 334.48 | 1154.85 | 3.45 |
| SM12261_0948 | Orf 9 protein | | 148.41 | 352.71 | 2.38 |
| SM12261_0950 | hypothetical protein | | 1.04 | 148.72 | 142.62 |
| SM12261_1098 | hypothetical protein | | 2508.30 | 19126.89 | 7.63 |
| SM12261_1099 | helix-turn-helix domain protein | | 1016.52 | 8914.00 | 8.77 |
| SM12261_1162 | peptide deformylase | | 4433.61 | 13180.83 | 2.97 |
| SM12261_1163 | hypothetical protein | | 42956.60 | 124660.35 | 2.90 |
| SM12261_1288 | hypothetical protein | | 7280.04 | 60952.94 | 8.37 |
| SM12261_1291 | periplasmic component of efflux system | | 82661.73 | 255808.54 | 3.09 |
| SM12261_1292 | ABC transporter. ATP-binding protein | | 43489.30 | 190901.90 | 4.39 |
| SM12261_1293 | ABC transporter permease protein | | 68795.66 | 414678.71 | 6.03 |
| SM12261_1313 | transposase | | 296.66 | 780.38 | 2.63 |
| SM12261_1388 | ComE operon protein 1 | | 526.23 | 62502.70 | 118.77 |
| SM12261_1389 | DNA internalization-related competence protein ComEC/Rec2 | | 4616.04 | 322871.28 | 69.95 |
| SM12261_1411 | competence protein | | 928.49 | 35003.27 | 37.70 |
| SM12261_1568 | DNA repair protein RadC | | 918.41 | 142161.88 | 154.79 |
| SM12261_1605 | DNA topoisomerase I | | 131712.41 | 525688.64 | 3.99 |
| SM12261_1606 | chlorohydrolase | | 1.04 | 6.09 | 5.84 |
| SM12261_1607 | DNA protecting protein DprA | | 2679.36 | 137715.01 | 51.40 |
| **Downregulated genes (>2-fold)** | |  |  |  |  |
| SM12261_0425 | alpha/beta hydrolase | | 1179.04 | 527.34 | -2.24 |
| SM12261_0432 | M protein trans-acting positive regulator (MGA) | | 321.52 | 0.97 | -331.46 |
| SM12261_0650 | hypothetical protein | | 312.48 | 143.67 | -2.17 |
| SM12261_0714 | chlorohydrolase | | 141.23 | 0.97 | -145.60 |
| SM12261_0771 | hypothetical protein | | 44.50 | 0.97 | -45.88 |
| SM12261_0957 | putative transcriptional regulator | | 91.90 | 26.58 | -3.46 |
| SM12261_0968 | protein LplB | | 1036.40 | 214.70 | -4.83 |
| SM12261_0995 | CpsH protein | | 12583.77 | 5972.20 | -2.11 |
| SM12261_0998 | Eps6K | | 2660.85 | 1148.99 | -2.32 |
| SM12261_1012 | hypothetical protein | | 29.04 | 10.47 | -2.77 |
| SM12261_1054 | translation elongation factor P | | 211.29 | 0.97 | -217.82 |
| SM12261_1235 | hypothetical protein | | 2618.40 | 597.08 | -4.39 |
| SM12261_1248 | hypothetical protein | | 156.94 | 43.61 | -3.60 |
| SM12261_1580 | high affinity ribose transport protein RbsD | | 2225.55 | 1052.46 | -2.11 |
| SM12261_1598 | hypothetical protein | | 26.23 | 0.97 | -27.04 |
