# Supplementary material for: High-resolution profiles of the Streptococcus mitis CSP signaling pathway reveal core and strain-specific regulated genes
Source: BMC Genomics. 2018 Jun 13;19:453. doi: 10.1186/s12864-018-4802-y (PMC6001120; doi:10.1186/s12864-018-4802-y)
Supplement: Supplementary file 2 — Table S2. Type strain genes up and downregulated (> 2-fold) in response to CSP in C + YYB. (DOCX 19 kb) [file 12864_2018_4802_MOESM2_ESM.docx]

Additional file 2: **Table S2.** Type strain genes up and downregulated (>2-fold) in response to CSP in C+Y_YB_.

| **Gene ID** | **Annotation** | **Mean A**  **(MIWT-Control)** | **Mean B (MIWT-CSP)** | **Fold Change (B/A)** |
| --- | --- | --- | --- | --- |
| **Upregulated genes (>2-fold)** | | | | |
| SM12261_0014 | SigX1 | 108.28 | 370.74 | 3.42 |
| SM12261_0025 | competence-induced protein Ccs16 | 314.56 | 2065.16 | 6.57 |
| SM12261_0026 | conserved hypothetical protein | 129.70 | 652.47 | 5.03 |
| SM12261_0044 | hypothetical protein | 7.43 | 943.70 | 126.93 |
| SM12261_0045 | hypothetical protein | 8.38 | 1591.77 | 190.02 |
| SM12261_0046 | bacteriocin-type signal sequence domain protein | 10.60 | 1825.20 | 172.26 |
| SM12261_0047 | hypothetical protein | 11.04 | 1818.16 | 164.70 |
| SM12261_0048 | ComA | 2041.89 | 17034.67 | 8.34 |
| SM12261_0049 | ComB | 2003.06 | 13902.76 | 6.94 |
| SM12261_0050 | phosphoribosylaminoimidazole-succinocarboxamide synthase | 6.94 | 30.22 | 4.36 |
| SM12261_0051 | phosphoribosylformylglycinamidine synthase | 36.57 | 103.02 | 2.82 |
| SM12261_0053 | phosphoribosylformylglycinamidine cyclo-ligase | 8.60 | 29.92 | 3.48 |
| SM12261_0054 | phosphoribosylglycinamide formyltransferase | 5.05 | 10.10 | 2.00 |
| SM12261_0056 | bifunctional purine biosynthesis protein PurH | 15.87 | 60.65 | 3.82 |
| SM12261_0240 | conserved hypothetical protein | 4.66 | 23.95 | 5.14 |
| SM12261_0241 | conserved domain protein | 1.72 | 28.71 | 16.69 |
| SM12261_0438 | type 4 prepilin peptidase | 9.99 | 569.80 | 57.05 |
| SM12261_0530 | heat-inducible transcription repressor HrcA | 1090.07 | 2211.24 | 2.03 |
| SM12261_0532 | chaperone protein DnaK | 4854.18 | 10234.71 | 2.11 |
| SM12261_0533 | 4-methyl-5 | 792.25 | 1871.79 | 2.36 |
| SM12261_0534 | chaperone protein DnaJ | 1314.53 | 3201.22 | 2.44 |
| SM12261_0542 | conserved hypothetical protein | 3.05 | 8.45 | 2.77 |
| SM12261_0543 | immunity protein BlpL | 10.43 | 27.64 | 2.65 |
| SM12261_0544 | conserved hypothetical protein | 6.88 | 16.50 | 2.40 |
| SM12261_0613 | SigX2 | 68.77 | 212.45 | 3.09 |
| SM12261_0628 | adenine-specific methyltransferase | 229.04 | 745.45 | 3.25 |
| SM12261_0629 | methyltransferase small domain superfamily | 122.25 | 9574.23 | 78.32 |
| SM12261_0630 | ComG operon protein 6 | 53.82 | 10095.92 | 187.59 |
| SM12261_0631 | competence protein | 61.87 | 10185.90 | 164.63 |
| SM12261_0632 | competence protein | 34.40 | 6612.99 | 192.23 |
| SM12261_0633 | competence protein CglB | 100.21 | 16282.99 | 162.48 |
| SM12261_0634 | putative ABC transporter subunit ComYA | 121.41 | 17734.49 | 146.08 |
| SM12261_0684 | conserved hypothetical protein | 162.83 | 2045.72 | 12.56 |
| SM12261_0685 | caax amino protease family | 223.26 | 7537.83 | 33.76 |
| SM12261_0686 | membrane protein%2C putative | 142.72 | 5336.99 | 37.39 |
| SM12261_0687 | conserved domain protein | 45.06 | 2096.63 | 46.53 |
| SM12261_0688 | conserved hypothetical protein | 23.81 | 1411.80 | 59.31 |
| SM12261_0717 | immunity protein | 1.28 | 3.68 | 2.89 |
| SM12261_0718 | BlpY | 2.55 | 15.63 | 6.12 |
| SM12261_0746 | M protein trans-acting positive regulator (MGA) | 542.86 | 1371.99 | 2.53 |
| SM12261_0749 | membrane protein%2C putative | 1267.22 | 7473.92 | 5.90 |
| SM12261_0750 | lipoprotein%2C putative | 1112.09 | 6257.03 | 5.63 |
| SM12261_0757 | ABC transporter substrate-binding protein | 249.99 | 933.62 | 3.73 |
| SM12261_0758 | ABC transporter%2C permease protein | 134.46 | 405.09 | 3.01 |
| SM12261_0759 | conserved hypothetical protein | 49.00 | 183.76 | 3.75 |
| SM12261_0760 | CbpD | 192.50 | 25884.13 | 134.46 |
| SM12261_0764 | ribosome-associated factor Y | 427.64 | 943.82 | 2.21 |
| SM12261_0765 | competence protein ComFA | 9.65 | 1092.41 | 113.16 |
| SM12261_0826 | single-strand binding protein family | 88.51 | 8727.47 | 98.61 |
| SM12261_0853 | competence-induced protein Ccs4 | 851.82 | 2719.37 | 3.19 |
| SM12261_0911 | ComM | 36.62 | 378.10 | 10.32 |
| SM12261_0912 | conserved hypothetical protein | 250.07 | 579.29 | 2.32 |
| SM12261_0913 | acetyltransferase%2C gnat family | 527.83 | 1131.65 | 2.14 |
| SM12261_0915 | CinA | 631.40 | 13724.47 | 21.74 |
| SM12261_0916 | RecA | 2698.57 | 14278.91 | 5.29 |
| SM12261_0917 | DNA-damage-inducible protein | 304.69 | 1346.56 | 4.42 |
| SM12261_0940 | ABC transporter ATP-binding protein - Na+ export | 1881.25 | 5136.51 | 2.73 |
| SM12261_0941 | ABC transporter membrane-spanning permease - Na+ export | 1940.88 | 5739.73 | 2.96 |
| SM12261_0959 | lipoprotein%2C putative | 1.28 | 5.03 | 3.94 |
| SM12261_0964 | hypothetical protein | 9.60 | 21.53 | 2.24 |
| SM12261_1022 | transporter YvqF | 187.75 | 412.69 | 2.20 |
| SM12261_1025 | DNA alkylation repair enzyme | 155.50 | 323.59 | 2.08 |
| SM12261_1098 | conserved hypothetical protein | 24.70 | 161.02 | 6.52 |
| SM12261_1099 | helix-turn-helix domain protein | 10.27 | 104.36 | 10.16 |
| SM12261_1162 | peptide deformylase | 35.23 | 95.78 | 2.72 |
| SM12261_1163 | conserved hypothetical protein | 336.91 | 927.53 | 2.75 |
| SM12261_1204 | conserved domain protein | 6.16 | 16.00 | 2.60 |
| SM12261_1252 | thiamine-phosphate pyrophosphorylase | 5.60 | 11.94 | 2.13 |
| SM12261_1288 | conserved domain protein | 59.10 | 417.83 | 7.07 |
| SM12261_1291 | periplasmic component of efflux system | 941.44 | 2385.98 | 2.53 |
| SM12261_1292 | ABC transporter%2C ATP-binding protein | 655.24 | 1878.14 | 2.87 |
| SM12261_1293 | ABC transporter permease protein | 1097.97 | 4010.45 | 3.65 |
| SM12261_1388 | ComEA | 7.43 | 913.31 | 122.84 |
| SM12261_1389 | ComEC | 48.55 | 4544.30 | 93.59 |
| SM12261_1411 | competence protein | 6.60 | 386.55 | 58.55 |
| SM12261_1511 | lactose-specific phosphotransferase enzyme IIA component | 6.49 | 13.21 | 2.03 |
| SM12261_1568 | radC | 10.82 | 1810.60 | 167.33 |
| SM12261_1605 | DNA topoisomerase I | 917.08 | 5411.10 | 5.90 |
| SM12261_1607 | DprA | 23.81 | 1519.88 | 63.85 |
| **Downregulated genes (>2-fold)** | | | | |
| SM12261_0015 | conserved hypothetical protein | 39.46 | 7.11 | -5.55 |
| SM12261_0093 | ABC transporter ATP-bindng protein - possibly multidrug efflux | 4.22 | 1.71 | -2.47 |
| SM12261_0096 | Orf 9 protein | 2.11 | 0.83 | -2.54 |
| SM12261_0305 | hypothetical protein | 5.16 | 1.71 | -3.02 |
| SM12261_0375 | phosphotransferase system sugar-specific EII component | 142.77 | 53.77 | -2.66 |
| SM12261_0426 | oxidoreductase%2C short chain dehydrogenase/reductase family | 5.88 | 2.60 | -2.26 |
| SM12261_0457 | galactokinase | 17.09 | 7.75 | -2.21 |
| SM12261_0477 | conserved hypothetical protein | 42.01 | 11.18 | -3.76 |
| SM12261_0499 | putative transcriptional regulator | 20.75 | 7.37 | -2.82 |
| SM12261_0571 | chlorohydrolase | 1.72 | 0.83 | -2.07 |
| SM12261_0609 | conserved hypothetical protein | 9.33 | 2.16 | -4.32 |
| SM12261_0612 | conserved hypothetical protein | 51.06 | 14.54 | -3.51 |
| SM12261_0650 | conserved hypothetical protein | 37.79 | 12.89 | -2.93 |
| SM12261_0720 | protein LplC | 13.71 | 4.63 | -2.96 |
| SM12261_0725 | conserved hypothetical protein | 7.88 | 3.12 | -2.53 |
| SM12261_0751 | hypothetical protein | 7.05 | 3.04 | -2.32 |
| SM12261_0842 | aminopeptidase P | 6.44 | 2.86 | -2.25 |
| SM12261_0948 | Orf 9 protein | 2.16 | 0.83 | -2.60 |
| SM12261_0956 | integrase/recombinase%2C phage integrase family | 3.78 | 1.21 | -3.12 |
