# Supplementary material for: High-resolution profiles of the Streptococcus mitis CSP signaling pathway reveal core and strain-specific regulated genes
Source: BMC Genomics. 2018 Jun 13;19:453. doi: 10.1186/s12864-018-4802-y (PMC6001120; doi:10.1186/s12864-018-4802-y)
Supplement: Supplementary file 3 — Table S3. SK321 genes upregulated (> 2-fold) in response to CSP in C + YYB. (DOCX 33 kb) [file 12864_2018_4802_MOESM3_ESM.docx]

Additional file 3: **Table S3.** SK321 genes upregulated (>2-fold) in response to CSP in C+Y_YB_.

| **Gene ID** | **Annotation** | **Mean A (SK321-Control)** | **Mean B (SK321-CSP)** | **Fold Change (B/A)** |
| --- | --- | --- | --- | --- |
| SMSK321_0027 | hypothetical protein | 648.52 | 10470.59 | 16.15 |
| SMSK321_0028 | CAAX amino terminal protease family protein | 2903.76 | 47828.25 | 16.47 |
| SMSK321_0031 | translation elongation factor P | 14.90 | 622.41 | 41.78 |
| SMSK321_0072 | helix-turn-helix family protein | 17.72 | 355.18 | 20.05 |
| SMSK321_0073 | 5-methyltetrahydropteroyltriglutamate-- homocysteine S-methyltransferase | 375.05 | 777.62 | 2.07 |
| SMSK321_0135 | cation diffusion facilitator family transporter family protein | 479.14 | 1435.64 | 3.00 |
| SMSK321_0137 | polypeptide deformylase family protein | 87.47 | 1493.70 | 17.08 |
| SMSK321_0138 | hypothetical protein | 744.94 | 12494.02 | 16.77 |
| SMSK321_0139 | hypothetical protein | 12.39 | 272.11 | 21.96 |
| SMSK321_0140 | conserved hypothetical protein | 169.82 | 788.04 | 4.64 |
| SMSK321_0152 | acetyltransferase family protein | 387.56 | 836.47 | 2.16 |
| SMSK321_0189 | FmtB protiein | 0.70 | 1.45 | 2.06 |
| SMSK321_0190 | hypothetical protein | 0.70 | 1.45 | 2.06 |
| SMSK321_0205 | hypothetical protein | 0.70 | 2.31 | 3.27 |
| SMSK321_0256 | putative relaxase | 2.29 | 13.58 | 5.94 |
| SMSK321_0257 | hypothetical protein | 0.70 | 5.81 | 8.25 |
| SMSK321_0260 | hypothetical protein | 0.70 | 1.45 | 2.06 |
| SMSK321_0270 | hypothetical protein | 1.00 | 2.65 | 2.66 |
| SMSK321_0275 | hypothetical protein | 0.70 | 1.45 | 2.06 |
| SMSK321_0286 | PilC | 71.61 | 3377.61 | 47.17 |
| SMSK321_0289 | MacA | 1983.18 | 14806.83 | 7.47 |
| SMSK321_0290 | ABC transporter family protein | 1300.89 | 12502.79 | 9.61 |
| SMSK321_0291 | permease family protein | 2130.78 | 26347.87 | 12.37 |
| SMSK321_0334 | hypothetical protein | 455.09 | 1192.65 | 2.62 |
| SMSK321_0337 | ComW | 26.60 | 287.29 | 10.80 |
| SMSK321_0338 | adenylosuccinate synthetase | 3419.03 | 22123.09 | 6.47 |
| SMSK321_0339 | tRNA-specific adenosine deaminase | 124.80 | 285.81 | 2.29 |
| SMSK321_0342 | DNA repair protein RadA | 2042.05 | 4793.74 | 2.35 |
| SMSK321_0343 | carbonic anhydrase family protein | 1122.61 | 2697.87 | 2.40 |
| SMSK321_0344 | hypothetical protein | 1480.82 | 3286.45 | 2.22 |
| SMSK321_0346 | hypothetical protein | 542.83 | 14171.76 | 26.11 |
| SMSK321_0351 | hypothetical protein | 36.79 | 3053.22 | 82.99 |
| SMSK321_0352 | hypothetical protein | 121.91 | 11183.89 | 91.74 |
| SMSK321_0353 | hypothetical protein | 45.78 | 4406.45 | 96.25 |
| SMSK321_0356 | conserved hypothetical protein | 303.57 | 618.17 | 2.04 |
| SMSK321_0362 | cell wall binding repeat family protein | 504.27 | 1959.31 | 3.89 |
| SMSK321_0364 | hypothetical protein | 0.70 | 1.45 | 2.06 |
| SMSK321_0396 | hypothetical protein | 0.70 | 1.45 | 2.06 |
| SMSK321_0421 | ComEA | 12.08 | 7584.68 | 627.88 |
| SMSK321_0422 | ComEC | 49.02 | 41150.17 | 839.39 |
| SMSK321_0423 | capsule polysaccharide export ATP-binding protein CtrD | 27.49 | 14516.65 | 528.03 |
| SMSK321_0424 | hypothetical protein | 46.84 | 23348.65 | 498.48 |
| SMSK321_0443 | PepB | 2663.57 | 9046.56 | 3.40 |
| SMSK321_0444 | O-methyltransferase family protein | 1004.29 | 3869.10 | 3.85 |
| SMSK321_0445 | foldase protein prsA | 2701.12 | 7265.05 | 2.69 |
| SMSK321_0451 | DNA primase | 3290.59 | 8722.07 | 2.65 |
| SMSK321_0452 | RNA polymerase sigma factor RpoD | 2484.15 | 7668.89 | 3.09 |
| SMSK321_0453 | conserved hypothetical protein | 770.85 | 2299.04 | 2.98 |
| SMSK321_0484 | hypothetical protein | 0.70 | 1.45 | 2.06 |
| SMSK321_0493 | large conductance mechanosensitive channel protein | 834.70 | 1957.39 | 2.35 |
| SMSK321_0515 | 6-phospho-beta-glucosidase bglA | 7.85 | 16.42 | 2.09 |
| SMSK321_0522 | hypothetical protein | 0.70 | 1.45 | 2.06 |
| SMSK321_0523 | hypothetical protein | 0.70 | 1.45 | 2.06 |
| SMSK321_0524 | hypothetical protein | 0.70 | 1.45 | 2.06 |
| SMSK321_0646 | RadC | 30.55 | 9090.99 | 297.56 |
| SMSK321_0674 | hypothetical protein | 45.58 | 195.54 | 4.29 |
| SMSK321_0678 | hypothetical protein | 1189.04 | 11988.03 | 10.08 |
| SMSK321_0679 | hypothetical protein | 1195.02 | 12442.60 | 10.41 |
| SMSK321_0680 | RibF | 1192.75 | 12972.40 | 10.88 |
| SMSK321_0688 | hypothetical protein | 0.70 | 1.45 | 2.06 |
| SMSK321_0689 | 3-isopropylmalate dehydrogenase | 500.89 | 1071.21 | 2.14 |
| SMSK321_0690 | 2-isopropylmalate synthase | 645.20 | 1802.50 | 2.79 |
| SMSK321_0691 | conserved hypothetical protein | 167.24 | 1682.32 | 10.06 |
| SMSK321_0692 | copper homeostasis protein cutC | 587.55 | 5821.23 | 9.91 |
| SMSK321_0693 | conserved hypothetical protein | 244.07 | 3186.49 | 13.06 |
| SMSK321_0694 | DNA topoisomerase I | 1364.04 | 32811.54 | 24.05 |
| SMSK321_0695 | DprA | 29.85 | 10127.87 | 339.32 |
| SMSK321_0718 | helix-turn-helix protein%2C YlxM/p13 family protein | 1.29 | 2.91 | 2.25 |
| SMSK321_0735 | putative transposase | 0.70 | 1.45 | 2.06 |
| SMSK321_0857 | choline binding protein F | 482.49 | 2073.49 | 4.30 |
| SMSK321_0914 | hypothetical protein | 2.53 | 12.73 | 5.04 |
| SMSK321_0926 | hypothetical protein | 0.70 | 1.45 | 2.06 |
| SMSK321_0982 | hypothetical protein | 0.70 | 1.45 | 2.06 |
| SMSK321_1050 | PilD | 9.79 | 6803.23 | 694.59 |
| SMSK321_1066 | phosphate transport system protein phoU | 0.70 | 1.45 | 2.06 |
| SMSK321_1144 | heat-inducible transcription repressor HrcA | 1986.57 | 7412.64 | 3.73 |
| SMSK321_1145 | protein GrpE | 885.16 | 2968.18 | 3.35 |
| SMSK321_1146 | chaperone protein DnaK | 6589.22 | 22090.34 | 3.35 |
| SMSK321_1147 | chaperone protein DnaJ | 1644.28 | 7081.81 | 4.31 |
| SMSK321_1184 | hypothetical protein | 311.89 | 31729.71 | 101.73 |
| SMSK321_1185 | DNA-directed RNA polymerase%2C delta subunit | 1061.67 | 10921.09 | 10.29 |
| SMSK321_1186 | hypothetical protein | 636.93 | 6013.67 | 9.44 |
| SMSK321_1193 | SsbB | 31.68 | 22110.68 | 697.93 |
| SMSK321_1194 | 10 kDa chaperonin | 193.19 | 1188.09 | 6.15 |
| SMSK321_1195 | chaperonin GroL | 2325.47 | 10184.89 | 4.38 |
| SMSK321_1196 | uncharacterized BCR%2C COG1636 family protein | 607.08 | 2567.48 | 4.23 |
| SMSK321_1197 | methyltransferase domain protein | 106.62 | 283.54 | 2.66 |
| SMSK321_1198 | HTH-type transcriptional regulator rgg | 35.11 | 109.31 | 3.11 |
| SMSK321_1204 | RmuC, Ccs50 | 1959.59 | 8449.63 | 4.31 |
| SMSK321_1251 | SigX2 | 29.89 | 2127.76 | 71.20 |
| SMSK321_1265 | AckA | 2702.93 | 12210.38 | 4.52 |
| SMSK321_1266 | hypothetical protein | 339.45 | 6706.99 | 19.76 |
| SMSK321_1267 | ComGG | 179.39 | 39173.15 | 218.36 |
| SMSK321_1268 | ComGF | 35.32 | 35042.03 | 992.01 |
| SMSK321_1269 | ComGE | 18.31 | 18607.67 | 1016.13 |
| SMSK321_1270 | ComGD | 33.72 | 28753.73 | 852.61 |
| SMSK321_1271 | ComGC | 21.71 | 22315.23 | 1027.66 |
| SMSK321_1272 | ComGB | 58.13 | 62719.50 | 1079.04 |
| SMSK321_1273 | ComGA | 60.65 | 56391.76 | 929.76 |
| SMSK321_1304 | sensory transduction protein lytT | 141.05 | 661.86 | 4.69 |
| SMSK321_1305 | hypothetical protein | 18.64 | 15939.47 | 854.97 |
| SMSK321_1306 | bacteriocin-type signal sequence domain protein | 19.79 | 13361.90 | 675.13 |
| SMSK321_1307 | hypothetical protein | 21.54 | 10652.81 | 494.48 |
| SMSK321_1308 | hypothetical protein | 41.13 | 15802.92 | 384.19 |
| SMSK321_1309 | hypothetical protein | 4.23 | 168.13 | 39.77 |
| SMSK321_1310 | ComA | 1291.92 | 53795.39 | 41.64 |
| SMSK321_1311 | ComB | 864.92 | 41781.03 | 48.31 |
| SMSK321_1312 | phosphoribosylaminoimidazole-succinocarboxamide synthase | 6.79 | 188.51 | 27.78 |
| SMSK321_1313 | phosphoribosylformylglycinamidine synthase | 68.87 | 829.39 | 12.04 |
| SMSK321_1314 | amidophosphoribosyltransferase | 26.57 | 264.58 | 9.96 |
| SMSK321_1315 | phosphoribosylformylglycinamidine cyclo-ligase | 35.81 | 145.03 | 4.05 |
| SMSK321_1344 | hypothetical protein | 0.70 | 1.45 | 2.06 |
| SMSK321_1345 | hypothetical protein | 0.70 | 1.45 | 2.06 |
| SMSK321_1346 | hypothetical protein | 0.70 | 1.45 | 2.06 |
| SMSK321_1396 | CAAX amino terminal protease family protein | 312.87 | 1136.46 | 3.63 |
| SMSK321_1397 | ABC transporter%2C ATP binding protein | 256.66 | 1275.43 | 4.97 |
| SMSK321_1398 | hypothetical protein | 118.48 | 641.27 | 5.41 |
| SMSK321_1399 | helix-turn-helix family protein | 39.34 | 202.43 | 5.15 |
| SMSK321_1400 | hypothetical protein | 47.69 | 240.82 | 5.05 |
| SMSK321_1418 | luciferase-like monooxygenase family protein | 471.62 | 941.99 | 2.00 |
| SMSK321_1425 | hypothetical protein | 0.70 | 2.65 | 3.76 |
| SMSK321_1443 | hypothetical protein | 0.70 | 1.45 | 2.06 |
| SMSK321_1450 | ACT domain protein | 0.70 | 1.45 | 2.06 |
| SMSK321_1478 | anaerobic ribonucleoside-triphosphate reductase activating protein | 192.87 | 486.99 | 2.52 |
| SMSK321_1479 | acetyltransferase family protein | 144.63 | 382.21 | 2.64 |
| SMSK321_1480 | hypothetical protein | 35.33 | 106.41 | 3.01 |
| SMSK321_1481 | anaerobic ribonucleoside-triphosphate reductase | 1053.63 | 3130.20 | 2.97 |
| SMSK321_1482 | hypothetical protein | 1222.25 | 16544.75 | 13.54 |
| SMSK321_1558 | hypothetical protein | 0.70 | 1.45 | 2.06 |
| SMSK321_1564 | hypothetical protein | 0.70 | 1.45 | 2.06 |
| SMSK321_1567 | hypothetical protein | 0.70 | 1.45 | 2.06 |
| SMSK321_1568 | hypothetical protein | 0.70 | 1.45 | 2.06 |
| SMSK321_1569 | hypothetical protein | 1.00 | 3.50 | 3.52 |
| SMSK321_1570 | hypothetical protein | 0.70 | 1.45 | 2.06 |
| SMSK321_1573 | hypothetical protein | 1.29 | 3.16 | 2.45 |
| SMSK321_1579 | chain A%2C Crystal Structure Of Thermotoga Maritima Alpha-Fucosidase | 354.42 | 805.18 | 2.27 |
| SMSK321_1595 | mga helix-turn-helix domain protein | 923.10 | 9423.89 | 10.21 |
| SMSK321_1598 | conserved hypothetical protein | 4557.24 | 33401.62 | 7.33 |
| SMSK321_1599 | hypothetical protein | 3693.97 | 26254.83 | 7.11 |
| SMSK321_1606 | putative thiamine biosynthesis protein | 26.43 | 2284.14 | 86.41 |
| SMSK321_1607 | putative membrane protein | 6.10 | 724.11 | 118.70 |
| SMSK321_1608 | hypothetical protein | 4.86 | 451.86 | 92.93 |
| SMSK321_1609 | CbpD | 172.13 | 78915.97 | 458.47 |
| SMSK321_1613 | YfiA | 728.00 | 3010.70 | 4.14 |
| SMSK321_1614 | ComFA | 12.89 | 7624.55 | 591.71 |
| SMSK321_1632 | phage infection protein | 51.06 | 145.23 | 2.84 |
| SMSK321_1634 | ComD | 1073.67 | 20298.17 | 18.91 |
| SMSK321_1638 | chromosomal replication initiator protein DnaA | 908.74 | 1825.30 | 2.01 |
| SMSK321_1651 | SigX1 | 53.61 | 1945.94 | 36.30 |
| SMSK321_1653 | ComM | 42.65 | 3215.20 | 75.38 |
| SMSK321_1654 | conserved hypothetical protein | 378.46 | 4131.41 | 10.92 |
| SMSK321_1655 | acetyltransferase family protein | 778.04 | 6190.52 | 7.96 |
| SMSK321_1656 | membrane-bound protein lytR | 1418.11 | 9821.21 | 6.93 |
| SMSK321_1657 | CinA | 925.06 | 51130.56 | 55.27 |
| SMSK321_1658 | protein RecA | 2360.44 | 44296.58 | 18.77 |
| SMSK321_1659 | MATE efflux family protein | 273.97 | 2957.01 | 10.79 |
| SMSK321_1660 | conserved hypothetical protein | 1059.84 | 2482.66 | 2.34 |
| SMSK321_1696 | ABC transporter family protein | 1390.96 | 17456.74 | 12.55 |
| SMSK321_1697 | hypothetical protein | 1455.11 | 18293.43 | 12.57 |
| SMSK321_1706 | hypothetical protein | 0.70 | 1.45 | 2.06 |
| SMSK321_1711 | hypothetical protein | 0.70 | 1.45 | 2.06 |
| SMSK321_1712 | hypothetical protein | 0.70 | 1.45 | 2.06 |
| SMSK321_1714 | hypothetical protein | 0.70 | 1.45 | 2.06 |
| SMSK321_1733 | ATP-dependent Clp protease ATP-binding subunit clpL | 1408.11 | 4899.48 | 3.48 |
| SMSK321_1757 | hypothetical protein | 22.46 | 83.76 | 3.73 |
| SMSK321_1758 | hypothetical protein | 22.37 | 76.52 | 3.42 |
| SMSK321_1759 | ComD | 124.49 | 603.44 | 4.85 |
| Downregulated genes | | | | |
| SMSK321_0998 | conserved hypothetical protein | 86.64958024 | 4.957142033 | 17.48 |
| SMSK321_0996 | ABC transporter%2C permease protein | 122.3738465 | 7.446591596 | 16.43 |
| SMSK321_0995 | putative oxidoreductase yjhC | 128.8705797 | 12.56566663 | 10.26 |
| SMSK321_0997 | bacterial extracellular solute-binding protein | 327.6994948 | 33.82505796 | 9.69 |
| SMSK321_0989 | conserved hypothetical protein | 25.98152394 | 4.449585948 | 5.84 |
| SMSK321_0225 | glutamine transport ATP-binding protein glnQ | 92.87256162 | 16.94516462 | 5.48 |
| SMSK321_0271 | stage V sporulation protein K | 27.46899296 | 5.556543162 | 4.94 |
| SMSK321_0224 | bacterial extracellular solute-binding protein%2C family 3 | 129.5590277 | 27.09146964 | 4.78 |
| SMSK321_1331 | mannose-specific phosphotransferase system component IIAB | 87.03797353 | 18.69985382 | 4.65 |
| SMSK321_1084 | cation diffusion facilitator family transporter | 191.8635396 | 41.43358256 | 4.63 |
| SMSK321_1524 | argininosuccinate synthase | 675.9174457 | 148.3033666 | 4.56 |
| SMSK321_0993 | ABC transporter sugar binding protein | 381.6213347 | 84.80072773 | 4.50 |
| SMSK321_1523 | argininosuccinate lyase | 496.6029988 | 110.4226766 | 4.50 |
| SMSK321_0227 | amino ABC transporter%2C permease protein His/Glu/Gln/Arg/opine family | 79.63298667 | 18.30589988 | 4.35 |
| SMSK321_1328 | PTS system%2C sorbose subfamily IIB component | 144.7782888 | 34.37612823 | 4.21 |
| SMSK321_1600 | hypothetical protein | 32.02782814 | 7.700369638 | 4.16 |
| SMSK321_0226 | amino ABC transporter%2C permease protein His/Glu/Gln/Arg/opine family | 115.9573623 | 28.1283389 | 4.12 |
| SMSK321_0009 | alkylhydroperoxidase AhpD family core domain protein | 54,35271524 | 13,67262384 | 3.98 |
| SMSK321_1330 | PTS system%2C mannose/fructose/sorbose family IID component | 302,6902944 | 79,10400867 | 3.83 |
| SMSK321_1525 | bacterial extracellular solute-binding protein%2C family 3 | 363,6172638 | 96,42618681 | 3.77 |
| SMSK321_1719 | conserved hypothetical protein | 9,985476274 | 2,65138256 | 3.77 |
| SMSK321_0583 | dihydroxyacetone kinase%2C L subunit | 249,2377433 | 71,56557203 | 3.48 |
| SMSK321_1329 | PTS system%2C sorbose-specific IIC component | 270,9476822 | 79,01216362 | 3.43 |
| SMSK321_1159 | hypothetical protein | 19,34791484 | 5,648388207 | 3.43 |
| SMSK321_0984 | bacitracin transport ATP-binding protein bcrA | 15,22938486 | 4,449585948 | 3.42 |
| SMSK321_0272 | hypothetical protein | 24,16769733 | 7,262901505 | 3.33 |
| SMSK321_1233 | IICman | 4487,109323 | 1351,039856 | 3.32 |
| SMSK321_0994 | putative N-acetylmannosamine-6-P epimerase | 149,5131073 | 45,05174643 | 3.32 |
| SMSK321_1232 | mannose-specific phosphotransferase system component IIAB | 3979,764309 | 1217,51607 | 3.27 |
| SMSK321_1058 | RegM | 6,70373435 | 2,051981431 | 3.27 |
| SMSK321_1275 | alcohol dehydrogenase | 353,4038923 | 110,5749763 | 3.20 |
| SMSK321_1234 | IIDman | 5657,967656 | 1800,779046 | 3.14 |
| SMSK321_1059 | protein SapB | 11,25447998 | 3,850184819 | 2.92 |
| SMSK321_1716 | protein LplB | 12,78443099 | 4,449585948 | 2.87 |
| SMSK321_1060 | putative ABC transporter permease protein y4fN | 29,36440897 | 10,25990715 | 2.86 |
| SMSK321_0606 | aldose 1-epimerase family protein | 24,03891098 | 8,461703764 | 2.84 |
| SMSK321_0584 | dihydroxyacetone kinase%2C DhaK subunit | 339,9633715 | 119,7762571 | 2.84 |
| SMSK321_0736 | choline binding protein J | 8,868187216 | 3,250783689 | 2.73 |
| SMSK321_1257 | aldehyde-alcohol dehydrogenase 2 | 3686,40687 | 1370,887855 | 2.69 |
| SMSK321_1061 | ABC transporter family protein | 29,09199751 | 11,27501932 | 2.58 |
| SMSK321_1332 | putative tagatose-6-phosphate ketose/aldose isomerase | 72,19832233 | 29,21353902 | 2.47 |
| SMSK321_1063 | conserved hypothetical protein | 10,54985288 | 4,357740903 | 2.42 |
| SMSK321_1231 | alcohol dehydrogenase family protein | 1266,590544 | 524,3235245 | 2.42 |
| SMSK321_0992 | lactose transport system permease protein LacF | 112,0701022 | 46,64450263 | 2.40 |
| SMSK321_1562 | primase C family protein | 6,341643947 | 2,65138256 | 2.39 |
| SMSK321_1603 | transcriptional regulator NadR | 180,4674687 | 75,64777779 | 2.39 |
| SMSK321_1155 | hypothetical protein | 4,862264485 | 2,051981431 | 2.37 |
| SMSK321_1512 | hypothetical protein | 49,25377211 | 21,02737039 | 2.34 |
| SMSK321_1540 | hypothetical protein | 755,0575214 | 322,5816123 | 2.34 |
| SMSK321_1321 | hypothetical protein | 18,95480659 | 8,207925722 | 2.31 |
| SMSK321_0844 | hypothetical protein | 4,640424585 | 2,051981431 | 2.26 |
| SMSK321_1728 | helix-turn-helix family protein | 26,68615105 | 11,8043325 | 2.26 |
| SMSK321_1082 | galactokinase | 28,78047866 | 12,81944467 | 2.25 |
| SMSK321_1541 | hypothetical protein | 1415,506223 | 630,5939074 | 2.24 |
| SMSK321_1445 | bacterial extracellular solute-binding family protein | 121,3266827 | 54,11285132 | 2.24 |
| SMSK321_1327 | beta-galactosidase | 490,3233906 | 219,8375482 | 2.23 |
| SMSK321_0991 | L-arabinose transport system permease protein AraQ | 91,14166477 | 41,43358256 | 2.20 |
| SMSK321_1437 | PTS system%2C Lactose/Cellobiose specific IIA subunit | 4,469156234 | 2,051981431 | 2.18 |
| SMSK321_1365 | hypothetical protein | 38,15100678 | 17,6146537 | 2.17 |
| SMSK321_1446 | ABC transporter family protein | 134,7961871 | 62,99026612 | 2.14 |
| SMSK321_0987 | ROK family protein | 93,78081529 | 44,10672221 | 2.13 |
| SMSK321_0988 | putative N-acetylneuraminate lyase | 100,5351212 | 47,77321694 | 2.10 |
| SMSK321_1542 | hypothetical protein | 1284,241261 | 613,6704999 | 2.09 |
| SMSK321_1322 | phosphoribosylaminoimidazole carboxylase%2C catalytic subunit | 60,7105383 | 29,14345107 | 2.08 |
| SMSK321_1052 | N-(5'-phosphoribosyl)anthranilate isomerase | 138,3355017 | 66,7268488 | 2.07 |
| SMSK321_1320 | phosphoribosylamine--glycine ligase | 208,8177659 | 100,8008681 | 2.07 |
| SMSK321_0184 | peptide methionine sulfoxide reductase MsrA/MsrB | 347,0372858 | 168,0787872 | 2.06 |
| SMSK321_1539 | transcriptional regulator PadR-like family protein | 340,4185155 | 167,3657839 | 2.03 |
| SMSK321_0081 | multiple sugar-binding transport ATP-binding protein MsmK | 1951,411815 | 975,3799353 | 2.00 |
