# Supplementary material for: High-resolution profiles of the Streptococcus mitis CSP signaling pathway reveal core and strain-specific regulated genes
Source: BMC Genomics. 2018 Jun 13;19:453. doi: 10.1186/s12864-018-4802-y (PMC6001120; doi:10.1186/s12864-018-4802-y)
Supplement: Supplementary file 4 — Table S4. CSP responses by the type strain and SK321 genes without orthologues in S. pneumoniae. (DOCX 13 kb) [file 12864_2018_4802_MOESM4_ESM.docx]

Additional file 4: **Table S4.** CSP responses by the type strain and SK321 genes without orthologues in *S. pneumoniae.*

| **Gene ID** |  |  | | **Mean fold-change** | |  |
| --- | --- | --- | --- | --- | --- | --- |
|  |  | **TSB** | | **C+Y_YB_** | |  |
| **NCTC12261**^a^ | **SK321** | | **NCTC12261** | **NCTC12261** | **SK321** | **Description^a^** |
| 0041 | 0857 | 1.2 | | 1.5 | 4.3 | Choline-binding protein F |
| 0749 | 1598 | 6.2 | | 5.9 | 7.3 | Membrane protein |
| 0750 | 1599 | 12.4 | | 5.6 | 7.1 | Lipoprotein, putative |
| 0044 | - | 232.5 | | 126.9 | - | Hypothetical protein |
| 0045 | - | 260.6 | | 190.0 | - | Bacteriocin-type signal sequence |
| 0046 | - | 195.2 | | 172.3 | - | Hypothetical protein |
| 0047 | - | 230.3 | | 164.7 | - | Hypothetical protein |
| 0240 | - | 24.4 | | 5.1 | - | Hypothetical protein |
| 0241 | - | 19.8 | | 16.7 | - | Hypothetical protein |
| - | 1305 |  | | - | 855.0 | Hypothetical protein |
| - | 1306 |  | | - | 675.1 | Bacteriocin-type signal sequence |
| - | 1307 |  | | - | 494.5 | Hypothetical protein |
| - | 1308 |  | | - | 384.2 | Hypothetical protein |
| - | 1309 |  | | - | 39.8 | Hypothetical protein |
| - | 1184 |  | | - | 101.7 | Hypothetical protein |
| - | 1186 |  | | - | 9.4 | Hypothetical protein |
