# Supplementary material for: High-resolution profiles of the Streptococcus mitis CSP signaling pathway reveal core and strain-specific regulated genes
Source: BMC Genomics. 2018 Jun 13;19:453. doi: 10.1186/s12864-018-4802-y (PMC6001120; doi:10.1186/s12864-018-4802-y)
Supplement: Supplementary file 5 — Table S5. Strains, mutants and primers used in this study. (DOCX 19 kb) [file 12864_2018_4802_MOESM5_ESM.docx]

Additional file 5: **Table S5**. Strains, mutants and primers used in this study.

| **Strains** | **Description** | **Source, use, or characteristics** |
| --- | --- | --- |
|  | *Genotype;* phenotype. Symbol for insertion is :: |  |
| CCUG 31611^T^ | *S. mitis* biovar 1 type strain, corresponds to NCTC 12261^T^; encapsulated, transformable strain; Kan^s^ Erm^s^ Spec^S^ | CCUG |
| NCTC 8033 (SK321) | *S. mitis* strain isolated from the oral cavity; Kan^s^ Erm^s^ Spec^S^ | NCTC[20] |
| NCTC7466 | *Streptococcus pneumoniae* D39 | NCTC |
| MI014 | CCUG 31611, but *sigX*, *sigX2*: Spec^R^, Kan^R^ | This study |
| MI042 | CCUG 31611, but SM12261_0044-0047: Kan^R^ | This study |
| MI055 | CCUG 31611, but p*_sigX_* luc::*spc*: Spec^R^ | [22] |
| MI092 | SK321, but p*_sigX_* luc::*spc*: Spc^R^ | [22] |
| MI123 | CCUG 31611, but p*_sigX_* luc::*spc*: Spec^R^, but SM12261_0044-0047: Kan^R^ | This study |
| MI142 | SK321, but SMSK321_1599: Kan^R^ | This study |
| MI143 | SK321, but SMSK321_1184: Kan^R^ | This study |
| MI144 | SK321, but SMSK321_1305-1309: Kan^R^ | This study |
| MI146 | SK321, but SMSK321_0857: Kan^R^ | This study |
| MI147 | CCUG 31611, but NCTC_0241: Kan^R^ | This study |
| MI149 | ΔNCTC_0041: Kan^R^ | This study |
| MI150 | ΔNCTC_0750: Kan^R^ | This study |
| **Primers** | **Sequence** |  |
| **Mutant construction** | | |
| FP001^a^ | ggcgcgccGTTTGATTTTTAATG | Kan^R^ cassette |
| FP068^b^ | ggccggccTAGGTACTAAAACAATTCATCCAGTA |  |
| FP395 | CGAATGCTCGTGTTGTTCAT | MI014 construction |
| FP396^a^ | ggcgcgcctaCAATCCCCTGGACTTCTTC |  |
| FP397^b^ | ggccggccTGAGGGAGGTATGTGGCTAGA |  |
| FP398 | AACGCAGGTCCATCTGGTAG |  |
| FP451^c^ | ggatccGATTGGGACCAAGAGGGAAT |  |
| FP452^d^ | gaattcCTCGGAAGCGTTCATGTCTT |  |
| FP437 | GCAGTGGACAAGAGGGAAAA | MI042 construction |
| FP686^a^ | ggcgcgccTTCTTCCGACGTTAGCTCAACAA |  |
| FP691^b^ | ggccggccGTACGGTCGTCATCTAGGAAG |  |
| FP692 | CGATAGTGACGTTTCCCAAA |  |
| FP1808 | CTTGGTTTATCTGCCTAG | MI142 construction |
| FP1809 | cattaaaaatcaaacAGTTGTTTCCTTTTTATTTCC |  |
| FP1810 | aaaaaggaaacaactGTTTGATTTTTAATGGATAATGTG |  |
| FP1811 | tcaaagcaacactggATCGATACAAATTCCTCG |  |
| FP1812 | ggaatttgtatcgatCCAGTGTTGCTTTGAGTCAATAATAAAATTTG |  |
| FP1813 | AATTCCGTGGCCTCGCTC |  |
| FP1886 | TGTTCAAGCAGGAGACGATG |  |
| FP1887 | GCTCCGTGGAGCTATTTCAC |  |
| FP1824 | GGCTTTGATGATGATCGTGG | MI143 construction |
| FP1825 | cattaaaaatcaaacGGCTCAAGCTAGCGAAGTTG |  |
| FP1826 | tcgctagcttgagccGTTTGATTTTTAATGGATAATGTG |  |
| FP1827 | cccatccgatggtgtATCGATACAAATTCCTCG |  |
| FP1828 | ggaatttgtatcgatACACCATCGGATGGGTAG |  |
| FP1829 | GACGATACCACACCACCAG |  |
| FP1890 | ATGATCGTGGCGATCTTTTC |  |
| FP1891 | GAACGACTGCACCAGATTCA |  |
| FP1856 | TCTTCACCTATAGTTTTGAGG | MI144 construction |
| FP1857 | cattaaaaatcaaacTGGTGCAATTCCTCCATC |  |
| FP1858 | ggaggaattgcaccaGTTTGATTTTTAATGGATAATGTG |  |
| FP1859 | taatcgaaaacagcgATCGATACAAATTCCTCG |  |
| FP1860 | ggaatttgtatcgatCGCTGTTTTCGATTATCGTTTTGTAC |  |
| FP1861 | TCGAAGCCAGCGCATCAATG |  |
| FP1892 | GGGCTTTACTCTGCCCTACC |  |
| FP1893 | TGTTAGCATCCGTGAAACGA |  |
| FP1848 | TACAAGGAAGGTAACACATG | MI146 construction |
| FP1849 | cattaaaaatcaaacAGCCATTTCCTCCTATTC |  |
| FP1850 | taggaggaaatggctGTTTGATTTTTAATGGATAATGTG |  |
| FP1851 | agattgcttatctatATCGATACAAATTCCTCG |  |
| FP1852 | ggaatttgtatcgatATAGATAAGCAATCTTCACAAC |  |
| FP1853 | TCTTGATTAGCTATGGCAG |  |
| FP1884 | CGCACAGGTTGGTACAAAGA |  |
| FP1885 | CCATACTGGCGACAATTCCT |  |
| FP1864 | GAAACTAATCTAGGAACAATGATTTTC | MI147 construction |
| FP1865 | cattaaaaatcaaacTTTCCATGGGGAATACCTC |  |
| FP1866 | tattccccatggaaaGTTTGATTTTTAATGGATAATGTG |  |
| FP1867 | ataaaacacctccagATCGATACAAATTCCTCG |  |
| FP1868 | ggaatttgtatcgatCTGGAGGTGTTTTATGAG |  |
| FP1869 | GAGTACTCTTGTAAATCACTG |  |
| FP1896 | AATCTAGGAACAATGATTTTCTCTTT |  |
| FP1897 | ACCACCTAGAATCGCAGCAC |  |
| FP1840 | GGTACTACCTCAAAGGTAG | MI149 construction |
| FP1841 | cattaaaaatcaaacCAGCGCCATTTTTAACCC |  |
| FP1842 | ttaaaaatggcgctgGTTTGATTTTTAATGGATAATGTG |  |
| FP1843 | tacttatttggctgtATCGATACAAATTCCTCG |  |
| FP1844 | ggaatttgtatcgatACAGCCAAATAAGTATGAATATTAC |  |
| FP1845 | TATCTTGATTAGCTATAGCAG |  |
| FP1880 | caactgcaaatggtcaatgg |  |
| FP1881 | tcctgcaaaatcatgacgaa |  |
| FP1872 | GCAAGGCTCACCCTTATC | MI150 construction |
| FP1873 | cattaaaaatcaaacTCATTTTTCTACTCCTTTGTAATATTTTAG |  |
| FP1874 | ggagtagaaaaatgaGTTTGATTTTTAATGGATAATGTG |  |
| FP1875 | aagcaccaaattttaATCGATACAAATTCCTCG |  |
| FP1876 | ggaatttgtatcgatTAAAATTTGGTGCTTTAACTTTTACTAGAAATC |  |
| FP1877 | GGTGCTGACGCAGTCATG |  |
| FP1894 | GCAAGGCTCACCCTTATCAA |  |
| FP1895 | CCTCTTCAACCAAATCAACCA |  |
| **Real-Time PCR** | | |
| FP369 | GCCGTTCGTGGTATGAGTCG | *gyrA* |
| FP370 | GGTCGCAACTGTGCGCTTAC |  |
| FP495 | GACAACGGGAAAAGTTCGTG | *comE* |
| FP496 | GCGTAAGGATTGTGATGACG |  |
| FP497 | CATCGTATCAGTGAGGGAGGT | *sigX* |
| FP498 | CTCGGAAGCGTTCATGTCTT |  |
| FP499 | GAGATTCGGGAGGACATTGA | *comA* |
| FP500 | ACGTTGACCACCTGAGATC |  |
| FP1020 | GATTTGTTGAGCTAACGTCGGAAG | SM12261_0044 |
| FP1021 | AAACCTCTACGAAATCCAATGTACCA |  |
| FP1022 | GCTAGTACCGAGGGCGGACTT | SM12261_0047 |
| FP1023 | TCTGCTTCCTAGATGACGACCGTA |  |
| FP1028 | TGTCATGGAGTTGGCAGAAGC | SM12261_0240 |
| FP1029 | CAACCTTTGGGCTCAATGATTTCT |  |
| FP1030 | TGCAGGGGAAGTTTGCACAT | SM12261_0241 |
| FP1031 | ACACCTCCAGCCCAAGTTCC |  |
| FP1034 | GGCCTACTGGCGGGTTAAGA | SM12261_0749 |
| FP1035 | TCCTGTTCCCAAAGCGATGA |  |
| FP1036 | CAACTGCAACGCCATCACAA | SM12261_0750 |
| FP1037 | TGGGCTTGAGGAGCTTGTTG |  |
| FP1038 | TCCTGAGGATGCGACTTCTCC | SM12261_0947 |
| FP1039 | TCTTTCGGGCATTTTCATGG |  |
| FP1040 | AGGATTTTGATCAGATATGCGAATACA | SM12261_0950 |
| FP1041 | CGCATGATACAAACGAGCCTTC |  |

^a^*AscI* restriction site underlined; ^b^*FseI* restriction site underlined; ^c^*Bam*HI restriction site underlined; ^d^*Eco*RI restriction site underlined. Lowercase letters not underlined correspond to overlapping regions for mutant construction.
